# Biodegradable nanoplatform upregulates tumor microenvironment acidity for enhanced cancer therapy via synergistic induction of apoptosis, ferroptosis, and anti-angiogenesis

**DOI:** 10.1186/s12951-023-01814-5

**Published:** 2023-02-22

**Authors:** Caiyun Zhang, Peng Wang, Ya’ nan Zhang, Pengpeng Lu, Xiaodan Huang, Yinfeng Wang, Lang Ran, Huan Xin, Xiaotong Xu, Wenjuan Gao, Yu Sun, Li Zhang, Guilong Zhang

**Affiliations:** 1 School of Pharmacy, Shandong Technology Innovation Center of Molecular Targeting and Intelligent Diagnosis and Treatment, Binzhou Medical University, Yantai, 264003 People’s Republic of China; 2 Institute of Aging Medicine, Binzhou Medical University, Yantai, 264003 Shandong China; 3 Department of Urology, the First Affiliated Hospital of Anhui Medical University, Institute of Urology, Anhui Medical University and Anhui Province Key Laboratory of Genitourinary Diseases, Anhui Medical University, Hefei, 230022 Anhui People’s Republic of China; 4 Key Laboratory of Tissue Microenvironment and Tumor, Shanghai Institute of Nutrition and Health, Chinese Academy of Sciences, University of Chinese Academy of Sciences, Shanghai, 200031 China; 5 Department of Medicine and VAPSHCS, University of Washington, Seattle, WA 98195 USA

**Keywords:** Biodegradable nanoplatform, Tumor microenvironment, Increased acidity, Starvation therapy, Enhanced chemodynamic therapy

## Abstract

Chemodynamic therapy of cancer is limited by insufficient endogenous H_2_O_2_ generation and acidity in the tumor microenvironment (TME). Herein, we developed a biodegradable theranostic platform (pLMOFePt-TGO) involving composite of dendritic organosilica and FePt alloy, loaded with tamoxifen (TAM) and glucose oxidase (GOx), and encapsulated by platelet-derived growth factor-B (PDGFB)-labeled liposomes, that effectively uses the synergy among chemotherapy, enhanced chemodynamic therapy (CDT), and anti-angiogenesis. The increased concentration of glutathione (GSH) present in the cancer cells induces the disintegration of pLMOFePt-TGO, releasing FePt, GOx, and TAM. The synergistic action of GOx and TAM significantly enhanced the acidity and H_2_O_2_ level in the TME by aerobiotic glucose consumption and hypoxic glycolysis pathways, respectively. The combined effect of GSH depletion, acidity enhancement, and H_2_O_2_ supplementation dramatically promotes the Fenton-catalytic behavior of FePt alloys, which, in combination with tumor starvation caused by GOx and TAM-mediated chemotherapy, significantly increases the anticancer efficacy of this treatment. In addition, T_2_-shortening caused by FePt alloys released in TME significantly enhances contrast in the MRI signal of tumor, enabling a more accurate diagnosis. Results of in vitro and in vivo experiments suggest that pLMOFePt-TGO can effectively suppress tumor growth and angiogenesis, thus providing an exciting potential strategy for developing satisfactory tumor theranostics.

**Supplementary Information:**

The online version contains supplementary material available at 10.1186/s12951-023-01814-5.

## Introduction

Malignant tumors are associated with unique microenvironments that feature weak acidity [1], overproduction of hydrogen peroxide (H_2_O_2_) [2], low catalase activity, and hypoxia [3]. Although these conditions are favorable for tumor growth and metastasis, they also present opportunities to target and treat these tumors. The atypical proliferation and metabolism of tumor cells result in the production of reactive oxygen species (ROS) [4]. Although limited ROS generation can facilitate tumor growth, high ROS concentrations can damage and kill the tumor cells [5]. H_2_O_2_ is one of the most important subtypes of ROS in cancer cells. It can also produce other kinds of ROS, such as the highly toxic hydroxyl radicals (·OH) via Fenton-type reactions [6]. The kinetics of these processes strongly depend on the existence of catalytic activity as well as on the local conditions such as pH and H_2_O_2_ concentration [7]. Previous reports show that the H_2_O_2_ concentration in tumor cells can reach 100 μM, which is four orders of magnitude higher than that in normal cells, around 10 nM [8]. This overproduction of H_2_O_2_ could be exploited to cause a localized increase in the concentration of ·OH radicals using Fenton catalysts, and form the basis of OH-mediated chemodynamic therapy (CDT) to selectively target and kill tumor cells while avoiding any significant damage to healthy, benign tissues [9]. However, the endogenous H_2_O_2_ overproduction and the relatively weakly acidic conditions present in the TME cannot generate sufficient ·OH to be effective as anti-tumor CDT [10]. Therefore, the development of high-performance Fenton catalysts and a way to increase the endogenous local H_2_O_2_ concentration and the acidity in the TME is critical for this strategy to be effective against tumors.

Ultrasmall iron oxide (USIO), a classical Fenton catalyst, has some inherent limitations such as low catalytic activity and the need for highly acidic conditions (with pH in the range of 2–4), making it unsuitable for achieving effective anti-tumor CDT [11]. Recent publications have reported that ultrasmall zero-valent nanoiron (ZVNI) shows higher Fenton catalytic activity than USIO in TME [12, 13]. However, ZVNI is easily oxidized at room temperature, which largely limits its ability to be used in CDT treatments. We believe that developing a stable ZVNI-based catalyst could be a very promising route to developing effective CDT treatments. FePt alloy as a stable zero-iron donor might be a great promising Fenton catalyst. Glucose oxidase (GOx) is a natural enzyme that catalyzes the conversion of glucose into gluconic acid and H_2_O_2_ in the presence of oxygen [14]. It is not only an H_2_O_2_ donor but also an acidity enhancer. Hence, one could logically deduce that the combination of Fenton catalysts and GOx could have significantly higher efficacy and anticancer activity as compared to Fenton catalysts alone. However, the hypoxic nature of the TME could likely limit the catalytic activity of GOx, resulting in less effective anti-cancer efficacy [15]. Recent research has shown that the drug tamoxifen (TAM), which is widely used in chemotherapy of estrogen receptor-positive (ER +) breast cancer, also activates the AMPK signal pathway and then lead to cellular non-oxygen-dependent glycolysis and lactate accumulation, which ultimately increases the cellular acidity [16, 17]. Therefore, we hypothesize that the co-delivery of TAM and GOx could alleviate the effect of hypoxia and insufficient acidity and boost the catalytic activity of Fe-based Fenton catalysts, improving their CDT performance. However, since uncontrolled delivery of GOx and TAM will damage normal tissues, the CDT system must have high specificity.

**Scheme 1:**
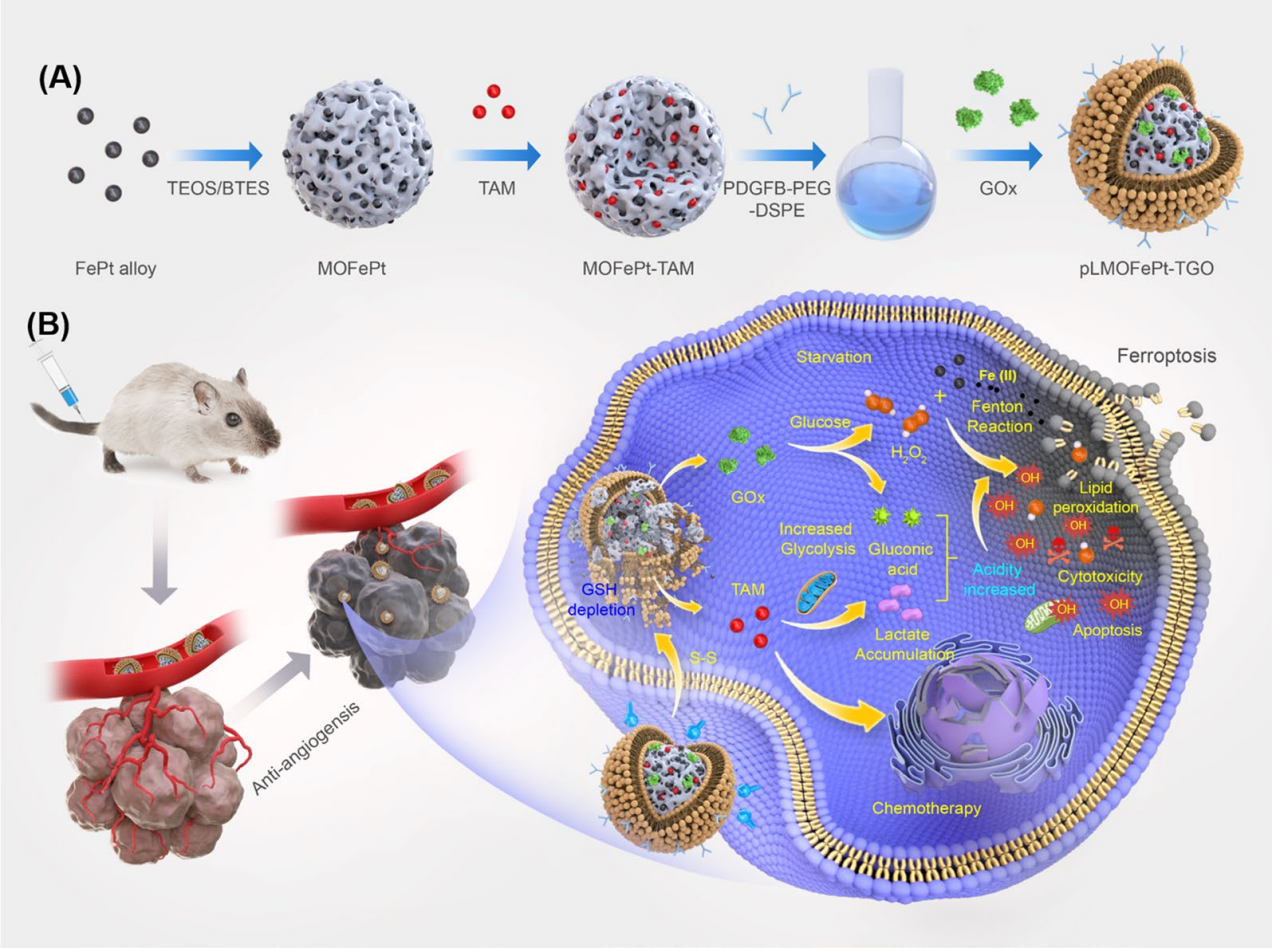
Schematic illustration of pLMOFePt-TGO preparation and its action mechanism of PDGFB-mediated tumor-targeted biodegradable nanoplatform to induce multipath cell death

We designed a biodegradable theranostic platform (pLMOFePt-TGO) that consists of TAM and GOx-loaded composite nanoparticles consisting of mesoporous organosilica (MON) and FePt alloy, encapsulated by platelet-derived growth factor-B (PDGFB)-labeled liposomes. As shown in, Scheme 1A, pLMOFePt-TGO was fabricated via the following steps: (1) disulfide-bridged mesoporous organosilica was decorated with FePt alloys to form MOFePt alloys; (2) TAM molecules were loaded in MOFePt alloys via physical absorption to form MOFePt-TAM; (3) MOFePt-TAM was encapsulated in PGDFB-labeled liposomes loaded with GOx to form the pLMOFePt-TGO nanoplatform. This nanoplatform is sensitive to cellular overproduction of glutathione (GSH), causing it to degrade, releasing FePt alloys, GOx, and TAM. The resulting GOx-mediated glucose consumption not only contributes to tumor starvation but also increases the cellular H_2_O_2_ levels and acidity. In addition, TAM contributes to chemotherapy and increases cellular acidity by triggering hypoxic glycolysis that results in increased lactate accumulation. The significantly increased acidity and H_2_O_2_ levels in the vicinity of the tumor, in turn, boost the Fenton catalytic activity of the FePt alloys, generating more hydroxyl radicals (·OH), thus enhancing anti-cancer CDT efficacy. In addition, the T_2_ shortening capability of ultrasmall FePt alloys facilitates better contrast in the T_2_-weighted MRI images of the tumor.

## Results and discussion

### Synthesis and characterization of pLMOFePt-TGO

**Fig. 1:**
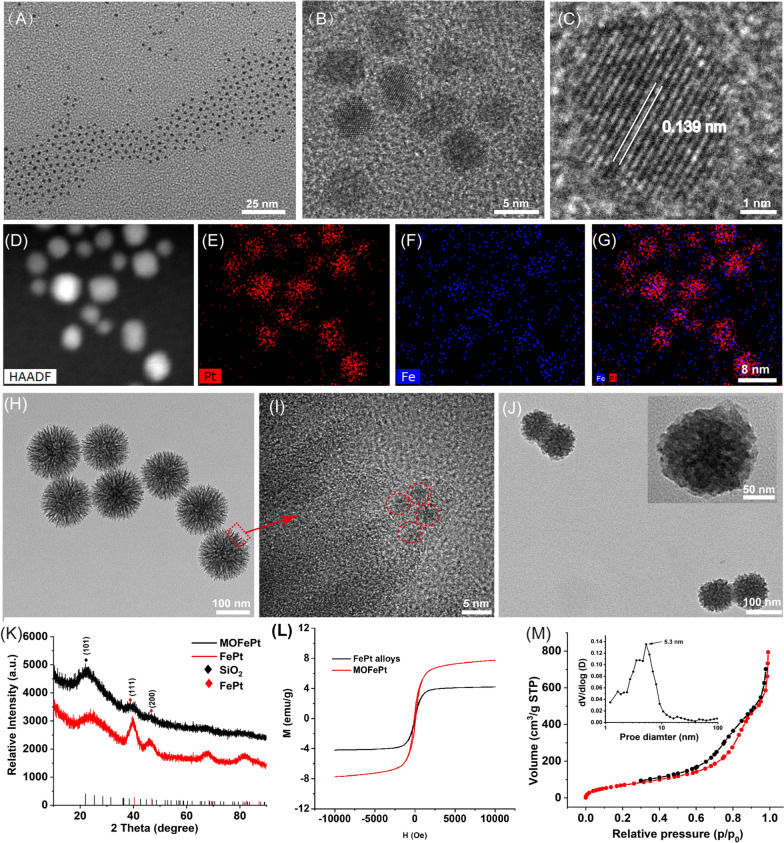
**A**–**C** TEM images of FePt alloys at different magnifications. **D**–**G** Elemental mapping images of FePt alloys. **H**–**I** TEM images of MOFePt at different magnifications. **J** TEM images of pLMOFePt-TGO. **K** XRD spectra and **L**, **M**–**H** curves of FePt alloy and MOFePt. **M** N_2_ adsorption/desorption isotherms of MOFePt

Ultrasmall FePt alloys were synthesized by a modified thermal decomposition method. Transmission electron microscope (TEM) images showed that the FePt alloys were 2–4 nm in size and had a narrow size distribution (Fig. 1A). High-resolution TEM (HRTEM) images showed clear lattice fringes with an interplanar spacing of 0.139 nm, corresponding to the (220) plane of the FePt alloy (Fig. 1B, C). Elemental mapping analysis further confirmed the presence of Fe and Pt, and the absence of O (Fig. 1D–G). These results confirmed that nanoparticles were composed of the FePt alloy. Subsequently, the FePt alloys were decorated into disulfide-bridged mesoporous organosilica to obtain MOFePt. It can be seen in Fig. 1H that the MOFePt alloys had a narrow size distribution centered around 150 nm. The structure of the MOFePt alloys was that of a dendritic sphere, with abundant pore channels. HRTEM images further revealed that the pore channels were decorated with large numbers of FePt alloys, implying the successful preparation of MOFePt alloys (Fig. 1I). Hydrodynamic size characterization revealed that while the FePt alloy had a narrow size distribution of approximately 2 nm, the MOFePt alloys were much larger, approximately 100 nm (Additional file 1: Fig. S1A), consistent with TEM observations.

Subsequently, the crystal structure of particles was characterized via X-ray diffraction (XRD). The XRD pattern (Fig. 1K) shows peaks at 23.37°, 40.31°, and 46.92°, corresponding to the (101), (111), and (200) planes, respectively, of the FePt alloys (PDF#29-0717). The XRD patterns of MOFePt alloys also show significant diffraction peaks at 23.37°, 40.31°, and 46.92°, consistent with the FePt alloy. However, the intensity of these peaks in MOFePt was significantly lower than that of the pure FePt alloy. This could be attributed to the shielding effect of MONs. This result further proved that the FePt alloys were successfully integrated into MON. Field-dependent magnetization (M-H) curves show that the saturated magnetization of MOFePt was 5.28 emu/g, while that of FePt alloys was only 41.72 emu/g (Fig. 1L). This was because the aggregation of FePt alloys in of MON increased the magnetization of MOFePt. Notably, the FePt and the MOFePt alloys showed the typical superparamagnetic behavior without magnetic hysteresis, which can be expected to enhance proton exchange and improve contrast during MRI. All these results confirmed that MOFePt was synthesized successfully as intended, with appropriate structure and morphology.

N_2_ adsorption-desorption isotherms indicated that the MOFePt alloys had a distinct mesoporous structure and a high specific surface area (272.95 m^2^/g). These nanoparticles have pore sizes primarily in the range of 2–10 nm and an average pore size of approximately 5.3 nm (Fig. 1M). These characteristics make the MOFePt alloys well-suited to function as drug carriers. TAM and GOx-loaded MOFePt alloys were encapsulated by PDGFB-labeled liposomes to fabricate the pLMOFePt-TGO theranostic agent. The hydrodynamic size of pLMOFePt-TGO increased from 150 nm as measured for MOFePt by themselves to approximately 220 nm after encapsulation (Additional file 1: Fig. S1A). Moreover, after the nanoparticle dispersions in various media were left undisturbed for 24 h, no significant change was observed in the hydrodynamic size of pLMOFePt-TGO nanoparticles, indicating excellent colloidal stability (Additional file 1: Figs. S1B, S2). The TEM image (Fig. 1J) shows the presence of a distinct membrane on the surface of the pLMOFePt-TGO. The full XPS spectrum of pLMOFePt-TGO presented in Additional file 1: Fig. S3 indicates the presence of the elements Fe, Pt, P, S, Si, C, and O. The high-resolution XPS spectra of pLMOFePt-TGO show Fe 2p peaks at 706.7 eV, and Pt 4f peaks at 74.1 eV and 70.4 eV, indicating the presence of zero-valent Fe and Pt. While, Fe(II) and Fe(III) peaks were also observed at 710.1 eV and 713.3 eV respectively, they could be attributed to some surface oxidation of the FePt alloys. In addition, S2p peaks can be seen at 163.4 eV, corresponding to the binding energy of the S–S bonded silicone. These results proved that pLMOFePt-TGO was successfully fabricated. In addition, the ζ potential of pLMOFePt-TGO was seen to be lower than that of MON and MOFePt, further verifying its successful fabrication (Additional file 1: Fig. S4). Fourier transform infrared spectroscopy (FT-IR) characterization of pLMOFePt-TGO showed absorption bands at 1400–1600 cm^−1^, 3500 cm^−1^ (benzene ring, R-NH_2_, TAM), and 2850–2930 cm^−1^ (–CH_2_, PEG-PDGFB) (Additional file 1: Fig. S5A). Furthermore, TGA results revealed that the loading rate of TAM in MOFePt-T was ~ 5.07% w/w (Additional file 1: Fig. S5B). Subsequently, based on UV standard curve of GOx and ICP analysis, the loading rate of GOx and FePt in pLMOFePt-TGO were 8.94% and 2.68%, respectively (Additional file 1: Fig. S5C, D).

### Cellular uptake and biodistribution of nanoplatforms

**Fig. 2:**
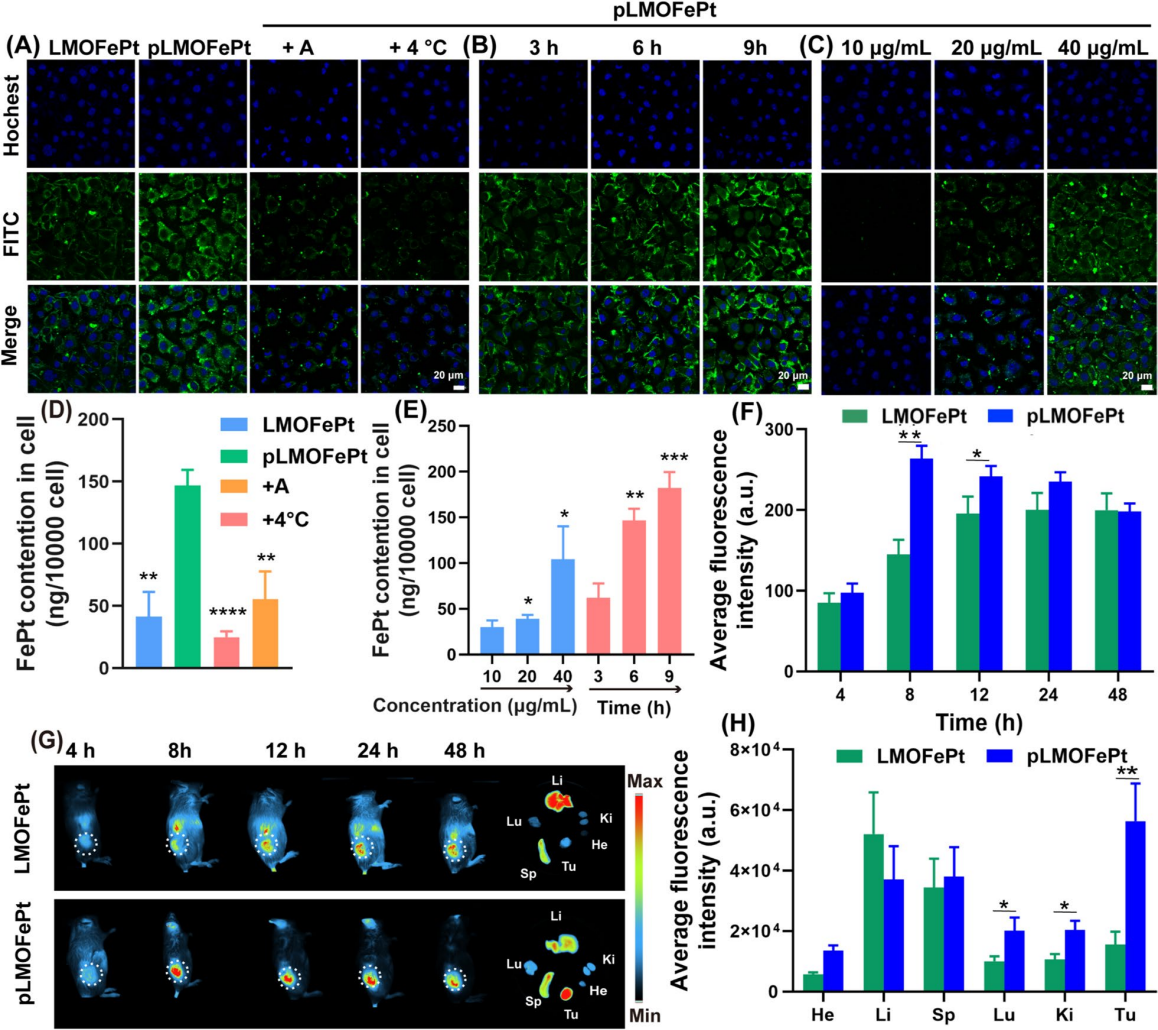
CLSM observation for internalization process: (A) treated with LMOFePt, pLMOFePt, pLMOFePt + amiloride, and pLMOFePt + 4℃ at the dosage of 40 μg/mL; (B) treated with pLMOFePt for different time at the dosage of 40 μg/mL; (C) treated with different concentrations of pLMOFePt for 4 h. ICP-MS quantitative analysis for internalization process: (D) treated with LMOFePt, pLMOFePt, pLMOFePt + amiloride, and pLMOFePt + 4℃ at the dosage of 40 μg/mL; (E) treated with different concentrations of pLMOFePt and pLMOFePt for different time at the dosage of 40 μg/mL (n=3, mean±SEM, *Means p < 0.05, **Means p < 0.01, ***Means p < 0.001). (F) Fluorescent intensity and (G) Fluorescent images of 4T1 tumor-bearing mice after injection of Cy7-labeled LMOFePt or Cy7-labeled pLMOFePt at the dosage of 10 mg/kg. (H) Fluorescent intensity of major organs and tumors in 4T1 tumor-bearing mice after injection Cy7-labeled LMOFePt or pLMOFePt at the dosage of 10 mg/kg for 48 h

The platelet-derived growth factor receptor (PDGFR) pathway is an important signaling network for the normal development of mesenchymal cells [18]. Overexpression of the PDGF-β receptor is one of the common features of various tumors that include breast cancer, cervical cancer, endometrial cancer, gastric cancer, and ovarian cancer [19–21]. Our previous work has shown that PDGFB ligands can be applied very effectively to target breast cancer-affected tissues [22]. Herein, we observed the internalization of pLMOFePt to assess its targeting ability via confocal laser microscopy (CLSM) and inductively coupled plasma-mass spectrometry (ICP-MS). LMOFePt and pLMOFePt were labeled with FITC to indicate the location of the nanoparticles within the tumor cells. As shown in Fig. 2A, the green fluorescence intensity from MCF-7 cells incubated with pLMOFePt was significantly stronger than those incubated with LMOFePt, indicating that PDGFB has an excellent ability to preferentially target MCF-7 cells. Remarkably, the green fluorescence of MCF-7 cells treated with pLMOFePt decreased significantly and was almost negligible in the presence of amiloride (an inhibitor of macropinocytosis), or at a low temperature (4 °C), suggesting that the internalization process of pLMOFePt was energy-mediated macropinocytosis. To further verify our findings, we used ICP-MS to quantitatively determine the FePt content in MCF-7 cells cultured with LMOFePt or pLMOFePt. The FePt content in pLMOFePt-treated MCF-7 cells was almost threefold that in LMOFePt-treated cells, further corroborating the excellent tumor selectivity of pLMOFePt. Moreover, cellular FePt content was observed to have reduced remarkably after co-incubation with amiloride or at a low temperature (Fig. 2D), which was consistent with CLSM observations. In addition, the fluorescence signal intensity of pLMOFePt-treated MCF-7 cells gradually increased with an increase in incubation time or dosage, revealing the cellular uptake of pLMOFePt was time- and dosage-dependent (Fig. 2B, C). Similarly, the change of nanoparticles quantitative result as determined by ICP-MS was consistent with CLSM observations (Fig. 2E). In addition, the internalization of pLMOFePt was also investigated in MCF-7 cells through bio-TEM observation (Additional file 1: Fig. S6C, D). Abundant pLMOFePt particles were observed in MCF-7 cells and part of pLMOFePt particles were significant degraded. Taken together, these findings support that PDGFB could increase the likelihood and the rate of MCF-7 cells taking in pLMOFePt by endocytosis.

We further explored the biodistribution and tumor-targeted accumulation of pLMOFePt in vivo. A 4T1 tumor-bearing mouse model was constructed by direct subcutaneous injection of 4T1 cells. After about a week, 4T1 tumor-bearing mice were monitored by an animal imaging system after intravenous injection (I.V.) of LMOFePt-Cy7 and pLMOFePt-Cy7. The fluorescence intensity of the tumor in mice injected with pLMOFePt-Cy7 gradually increased with time from 4 to 24 h, and then gradually decreased. It can be seen in Fig. 2F, that the tumor signal in mice treated with pLMOFePt-Cy7 reached the maximum after 8 h and was significantly stronger than that in mice treated with LMOFePt-Cy7. Figure 2G visually shows this phenomenon. These mice were subsequently euthanized, and the major organs and tumors were excised and observed using an animal imaging system. As shown in Fig. 2H, the fluorescence intensity of the tumor in mice treated with pLMOFePt-Cy7 was more than 3.6-fold that in mice treated with LMOFePt-Cy7, thus corroborating PDGFB-mediated tumor-targeting capability in vivo. The biodistribution study indicated that both LMOFePt-Cy7 and pLMOFePt-Cy7 primarily accumulated in the liver and the spleen. Additional file 1: Fig. S7A shows that pLMOFePt-Cy7 had a longer blood retention time compared to FePt alloys by themselves. This result demonstrated that our strategy for prolonging the blood circulation time of FePt alloys was successful, which would allow more time for the drug to accumulate in the tumor, thereby enhancing the anti-tumor activity of this nanoplatform.

### TME-dependent degradation and Fenton catalysis characteristics of MOFePt

**Fig. 3:**
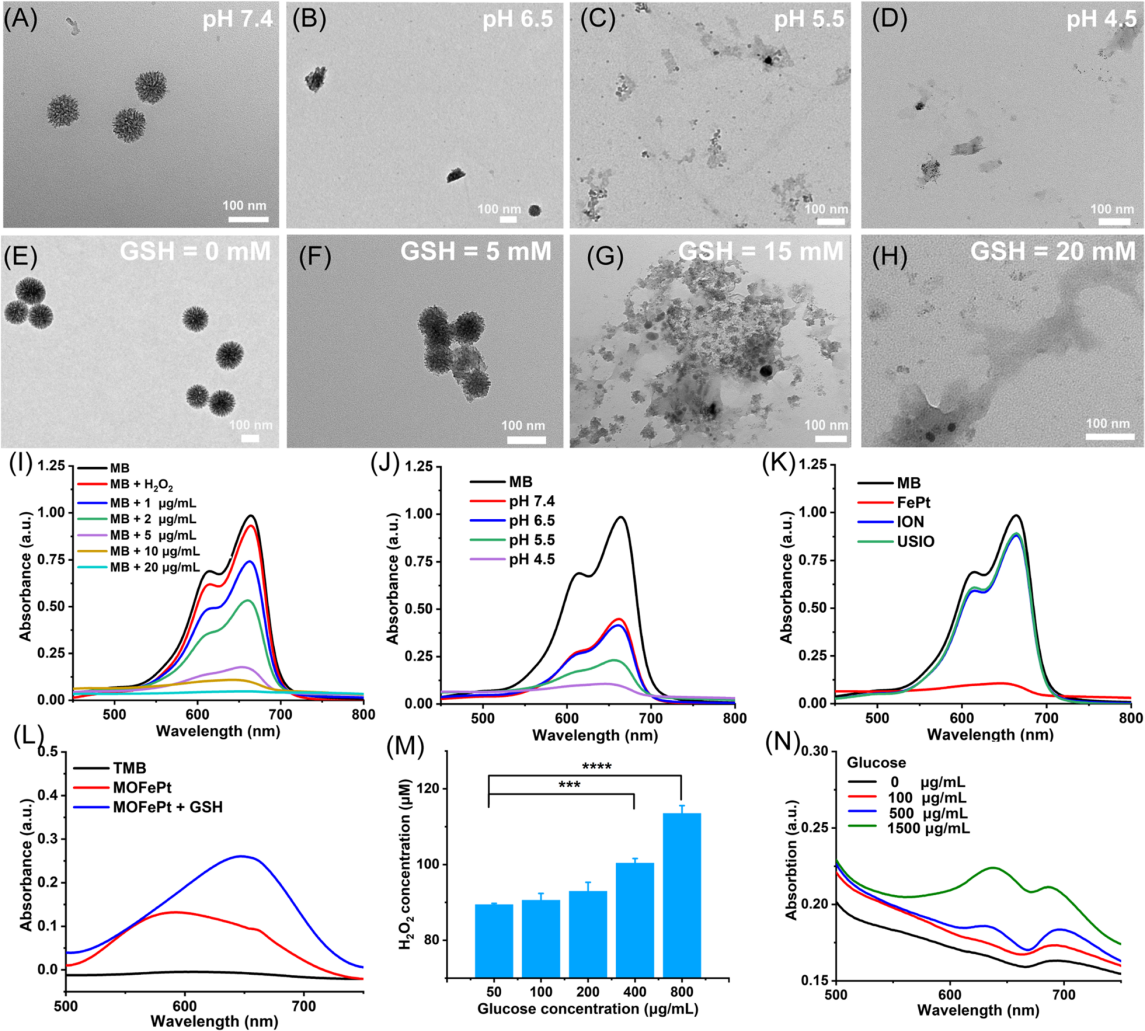
TEM images of MOFePt degradation at various (**A-D**) pH values and (**E-H**) GSH concentrations for 12 h. Degradation curves of MB solution treated with FePt alloy and H2O2 (10 mM): (**I**) different concentrations of FePt alloys; (**J**) different pH buffers (FePt: 5 μg/mL); (**K**) different samples (FePt: 10 μg/mL). (**L**) TMB color assay of MOFePt at absence and presence of GSH (5 mM). (**M**) H2O2 production of glucose solution after pLMOFePt-TGO (20 μg/mL) treatment. (**N**) TMB color assay of glucose solution treated with pLMOFePt-TGO (20 μg/mL) (mean±SEM, *Means p < 0.05, **Means p < 0.01, ***Means p < 0.001.

MOFePt alloys were exposed to phosphate buffer solutions with different pH values to simulate normal physiological conditions and TME. As shown in the TEM images, the structure of MOFePt degraded significantly in low pH conditions (Fig. 3A–D). Numerous free FePt alloys were observed around collapsed fragments after exposure to a pH of 4.5. In addition, we also investigated the biodegradability of MOFePt in response to GSH because of the possibility of the disulfide bonds in the MONs framework being cleaved by reduced GSH. As expected, the degradation of the MOFePt framework was seen to increase in severity with increasing GSH concentration (Fig. 3E–H). Along with the degradation, FePt alloys were gradually released from MOFePt and the release behavior shows pH-dependent relation (Additional file 1: Fig. S6B). The above results show that the degradation of MOFePt was indeed dependent on both the pH as well as the GSH concentration. This supports our proposed strategy to achieve tumor-specific theranostics using the pLMOFePt-TGO nanoplatform that gets selectively activated in TMEs.

Ferrous ions can catalyze the transformation of hydrogen peroxide (H_2_O_2_) into highly toxic hydroxyl radicals (·OH) via Fenton catalysis [23]. We next explored whether the FePt alloys released from MOFePt could catalyze the Fenton reaction. The degradation of methylene blue (MB) was used to assess the degree of ·OH production. First, we investigated the Fenton-catalytic activity of FePt alloy. As shown in Fig. 3I, J, MB solutions treated with FePt alloys degraded quickly and showed concentration and time-dependent variation. As compared to traditional ultrasmall iron oxide (USIO) and iron oxide nanoparticles (ION), FePt alloy showed a stronger ability to degrade MB, indicating that the FePt alloys generated more hydroxyl radicals, and thus were superior Fenton catalysts (Fig. 3K). The ability of MOFePt to catalyze Fenton reactions and produce ·OH was also detected via a 3, 3′, 5, 5′-tetramethylbenzidine (TMB) color assay. As shown in Fig. 3L, the UV absorbance of the H_2_O_2_ solution treated with MOFePt and 10 mM of GSH at 650 nm was significantly higher than that without the addition of GSH. This indicated that the rate of MOFePt catalyzed Fenton reaction is enhanced in the presence of GSH and produced more ·OH as a result. The ability of PLMOFePt-TGO to catalyze the transformation of glucose into gluconic acid and H_2_O_2_ was also assessed using an H_2_O_2_ assay kit and pH analyzer (Fig. 3M and Additional file 1: Fig. S6A). It could be seen that the addition of glucose solution generated significant amounts of H_2_O_2_, and the acidity of the solution increased gradually after pLMOFePt-TGO treatment. Moreover, the increase in H_2_O_2_ production and acidity were glucose concentration dependent. In addition, the TMB color assay indicated the UV absorbance at the wavelength of 650 nm gradually increased with the increase of glucose concentrations in the presence of pLMOFePt-TGO, indicating the ability of pLMOFePt-TGO to produce H_2_O_2_ and further induce ROS production (Fig. 3N). These results demonstrated that pLMOFePt-TGO has immense potential to improve the efficacy of CDT by self-supplying H_2_O_2_ and enhancing acidity.

### Mechanism of enhanced ROS production in vitro

**Fig. 4:**
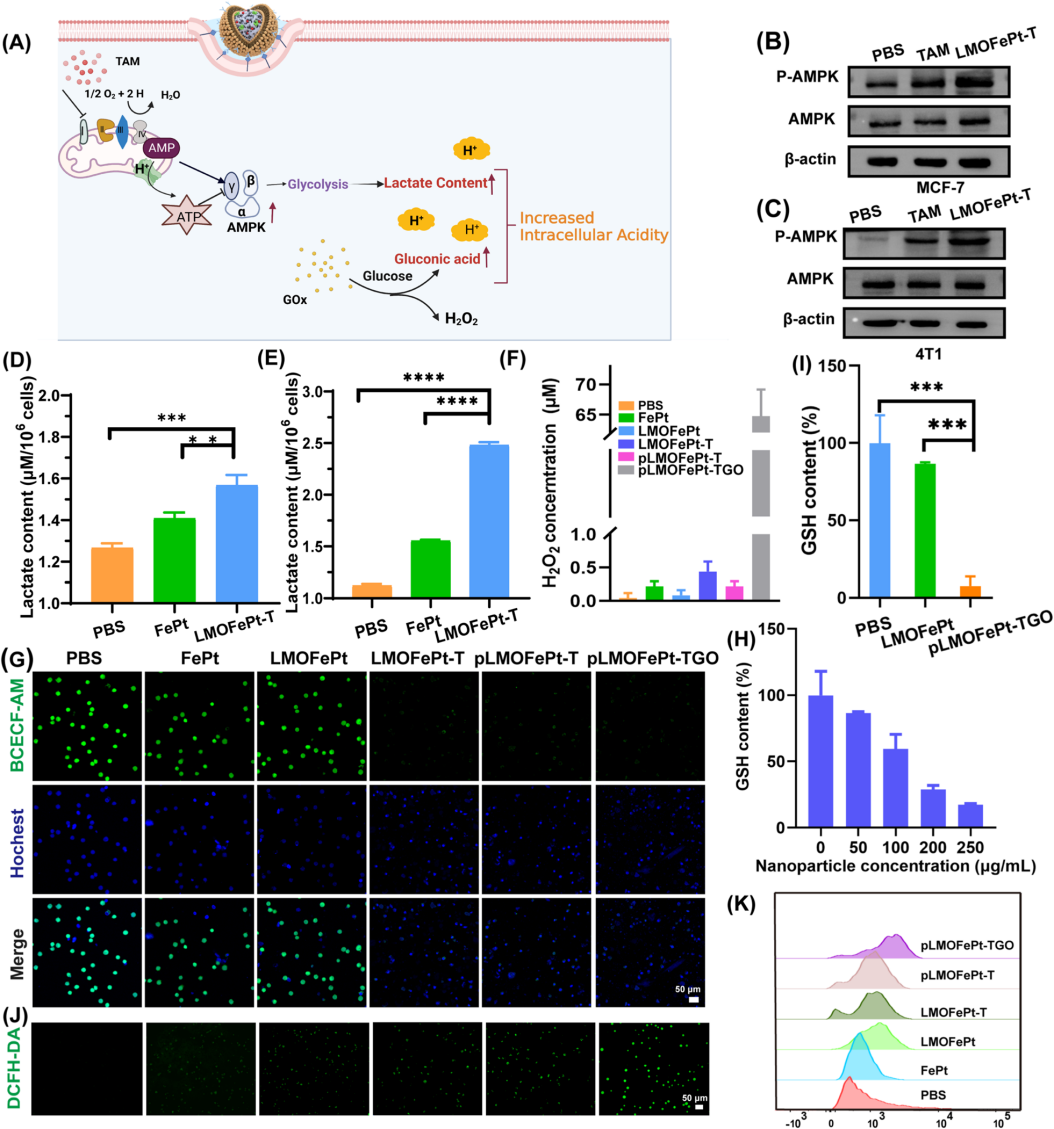
**A** Schematic diagram of mechanism of intracellular acidity enhancement. Determination of intracellular p-AMPK protein levels in **B** MCF-7 cells and **C** 4T1 cells by western blotting assay. Intracellular lactate content of **D** MCF-7 and **E** 4T1 cells incubated with PBS, FePt alloys, and LMOFePt-T at 5 μg/mL (equivalent Fe) for 12 h. **F** H_2_O_2_ content of MCF-7 cells treated with different samples at the concentration of 5 μg/mL (equivalent Fe) for 12 h. **G** CLSM observation for the acidity of MCF-7 cells stained by BCECF-AM after different samples at the concentration of 5 μg/mL (equivalent Fe) for 12 h. **H** GSH level of MCF-7 cells incubated with different concentrations of pLMOFePt-TGO. **I** GSH level of MCF-7 cells incubated with LMOFePt-TGO and pLMOFePt-TGO at the concentration of 20 μg/mL for 24 h. ROS production of MCF-7 cells stained by DCFH-DA after treatment with different samples for 4 h: **J** CLSM observation; **K** representative flow cytometric analysis. For **D**, **E** and **I**, data are means ± SD, n = 3, *P < 0.05; **P < 0.01; ***P < 0.005; ****P < 0.001

A previous study reported that TAM can inhibit the mitochondrial complex I [24], leading to an increase in the ratio of adenosine monophosphate (AMP) to adenosine triphosphate (ATP), thereby triggering the AMP-activated protein kinase (AMPK) signaling pathway [25], which is the major regulator of cellular energy homeostasis (Fig. 4A). AMPK can promote glycolysis, resulting in cellular lactate accumulation [16]. To verify the glycolysis process, the expressions of AMPK and p-AMPK in MCF-7 cells and 4T1 cells were analyzed by western blotting after TAM and pLMOFePt-TG treatment. It could be seen that the expression of p-AMPK in 4T1 and MCF-7 cells increased significantly in TAM and pLMOFePt-T treated groups (Fig. 4B, C). pLMOFePt-T was observed to have a stronger ability to induce p-AMPK expression than free TAM. These findings provided solid evidence in support of the hypothesis that pLMOFePt-T can lead to impaired mitochondrial oxidative phosphorylation. Intracellular lactate accumulation was measured using a lactate detection kit. As expected, the lactate content of the pLMOFePt-TGO treated cells was significantly higher than those treated with FePt alloys just by themselves as well as those treated with just PBS, particularly in hypermetabolic 4T1 tumor cells (Fig. 4D, E). The acidity of MCF-7 cells treated with different samples using a pH fluorescent probe 2*'*,7*'*-bis-(2-carboxyethyl)-5-(and-6)-carboxyfluorescein, an acetoxy-methyl ester (BCECF-AM) whose green fluorescence weakens in acidic conditions. The green fluorescence intensity of MCF-7 cells treated with FePt alloy by themselves as well as that of cells treated with LMOFePt was seen to be similar to that of the MCF-7 cells from the control group. However, the fluorescence intensity of the cells treated with LMOFePt-T and pLMOFePt-T was significantly weaker than the control group, with pLMOFePt-T treated cells showing this effect to a greater degree than the cells treated with LMOFePt-T without PDGFB labeling. These results proved that pLMOFePt-T could effectively enhance cellular acidity via the glycolysis process. In addition, the acidity of MCF-7 cells treated with pLMOFePt-TGO was higher than those treated with pLMOFePt-T, which could be attributed to the synergistic actions of GOx and TAM for enhancing cellular acidity (Fig. 4G). Subsequently, the cellular pH values were quantitatively analyzed via linear fitting of fluorescence intensity (Additional file 1: Fig. S7B, C). After treatment with pLMOFePt-TGO, the pH values of the cytoplasm in MCF-7 cells were around 4.0, which was significantly lower than that in the untreated cancer cells. While the limited H_2_O_2_ content in tumor regions can restrict the efficacy of the Fenton reactions [26, 27], GOx can convert intracellular glucose to produce gluconic acid and H_2_O_2_, thus increasing the acidity locally [28]. This led us to hypothesize that the upregulation of lactate and gluconic acid and the supplementation of H_2_O_2_ in cells would dramatically increase CDT efficacy. We first investigated intracellular H_2_O_2_ levels after different sample treatments. As compared to the cells in other groups, the H_2_O_2_ content of MCF-7 cells treated with pLMOFePt-TGO was significantly higher, indicating that pLMOFePt-TGO effectively elevated endogenous H_2_O_2_ concentration in cancer cells (Fig. 4F). Therefore, we concluded that pLMOFePt-TGO supplied abundant endogenous H_2_O_2_ as well as enhanced cellular acidity by the synergistic action of TAM-mediated non-oxygen glycolysis and the oxidation of glucose by GOx, significantly enhancing the Fenton catalytic activity and increasing the anti-cancer efficacy of CDT. It is well known that GSH is an antioxidant that reduces the ROS concentration and weakens the anti-cancer efficacy of CDT. Our results show that pLMOFePt-TGO could effectively tackle this challenge by consuming GSH because of the presence of disulfide bonds. We investigated cellular GSH levels after different sample treatments. As shown in Fig. 4H, while LMOFePt significantly decreased cellular GSH, pLMOFePt-TGO showed the strongest ability to reduce cellular GSH (Fig. 4I). Subsequently, we further investigated ROS generation in MCF-7 cancer cells using a 2′,7′-dichlorodihydrofluorescein diacetate (DCFH-DA) probe. As shown in Fig. 4J, the green fluorescence of MCF-7 cells treated with FePt or LMOFePt alone increased slightly, while cells treated with LMOFePt-T and pLMOFePt-T showed a significant increase in the intensity of green fluorescence. Notably, the pLMOFePt-TGO-treated cells showed the strongest green fluorescence, indicating a dramatic increase in cellular ROS production. Meanwhile, these results were also further confirmed by flow cytometry (Fig. 4K). These results demonstrated that pLMOFePt-TGO enhanced endogenous H_2_O_2_ levels and intracellular acidity in tumor cells, as well as consumed intracellular GSH, and as a result, significantly amplified the Fenton reaction, producing abundant ROS.

### In vitro *antitumor mechanism study*

**Fig. 5:**
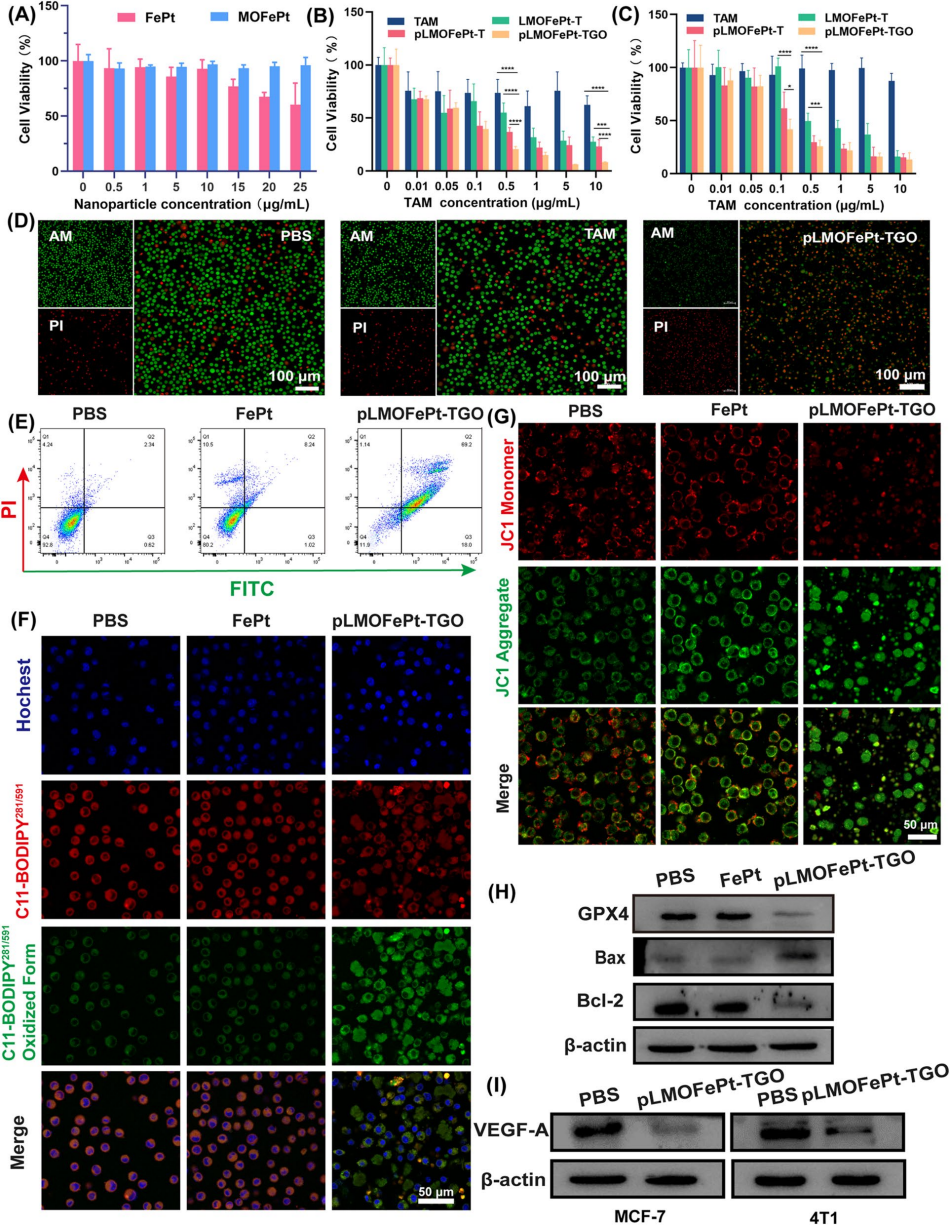
Viability of **A** THLE-3, **B** MCF-7, and **C** 4T1 cells treated with different samples. **D** Live/dead staining of MCF-7 cells treated different samples (equivalent TAM: 5 µg/mL). **E** Flow cytometry analysis for the apoptosis of MCF-7 cells at the different treatments (equivalent FePt: 5 µg/mL). **F** CLSM observation for the LPO of MCF-7 cells treated with different samples (equivalent FePt: 5 µg/mL). **G** CLSM observation for mitochondria damage of MCF-7 cells treated with different samples (equivalent FePt: 5 µg/mL). **H**–**I** Western blot analysis for the protein expression of Bcl-2, Bax, GPX4, and VEGF-A at different sample treatments (equivalent FePt: 5 µg/mL). For **B** and **C**, data are means ± SD. n = 5, *P < 0.05; **P < 0.01; ***P < 0.005; ****P < 0.001

The viability of cells was investigated by MTT assay. As compared to FePt alone, MOFePt did not cause a decrease in cell viability, showing lower cytotoxicity to the THLE-3 cells. This result shows that the MOFePt nanocarriers have excellent biocompatibility (Fig. 5A). In addition, the anti-cancer efficacy of pMOFePt-TGO against MCF-7 and 4T1 cells was assessed at different dosages. As shown in Fig. 5B, C, all samples showed a concentration-dependent variation in cell viability. Although TAM showed a certain degree of inhibition on MC-7 cells, it did not show any toxicity toward 4T1 cells. This was because TAM only selectively targeted estrogen receptor-positive (ER^+^) cells. Notably, while both LMOFePt-T and pLMOFePt-T exhibited a greater degree of inhibition on the viability of MCF-7 and 4T1 cells as compared to TAM, the cytotoxicity of pLMOFePt-T was stronger than that of LMOFePt-T without PDGFB labeling. These results demonstrated that the anti-cancer activity pLMOFePt-T was independent of the presence or absence of estrogen receptors and was strongly associated with glycolysis-enhanced CDT. As expected, pLMOFePt-TGO treatment exhibited the strongest anti-cancer inhibitory effect as compared to the other groups, indicating that the GOx-mediated starvation therapy further enhanced the efficacy of the ant-cancer CDT using pLMOFePt-TGO. In order to confirm the presence of ferroptosis, we explored the viability of MCF-7 cells treated with pLMOFePt-TGO at the absence and presence of iron ion chelate (DFOM) or ferroptotic inhibitor (Fer-1). As shown in Additional file 1: Fig. S8, the viability of MCF-7 cells significantly recovered after DFOM and Fer-1 treatment, further indicating the presence of ferroptosis. In addition, we also found that the use of NAC and apoptotic inhibitor also significantly recovered the viability of MCF-7 cells, indicating the presence of ROS-induced cell death and apoptosis. These results demonstrated that pLMOFePt-TGO-mediated cell death is derived from ferroptosis and apoptosis. The live/dead cell staining assay further confirmed that the anti-cancer activity of pLMOFePt was the most potent among the treatments tested in this study tumor cells (Fig. 5D).

The quantitative flow cytometric results shown in Fig. 5E indicated that the apoptotic/necrotic proportion of the cells treated with pLMOFePt-TGO was 88.34%. Iron ion-mediated Fenton reaction induces excessive ROS production in cells, which would trigger ferroptosis [29]. The expression of glutathione peroxidase 4 (GPX4) as a ferroptotic marker in MCF-7 cells was assessed after various treatments by western blotting. Significant downregulation of GPX4 was observed in the pLMOFePt-TGO group, suggesting that pLMOFePt-TGO induced ferroptosis (Fig. 5H). To verify pLMOFePt-TGO-induced ferroptosis, we further detected the intracellular lipid hydroperoxides (LPO) using the BPDIPY probe via CLSM observation. As shown in Fig. 5F and Additional file 1: Fig. S9B, the green fluorescence of MCF-7 and 4T1 cells incubated with the pLMOFePt-TGO was significantly stronger than those of the cells incubated in PBS or FePt alloy, clearly supporting the presence of LPO in cells. Hence, we concluded that the abundant ROS generated by pLMOFePt-TGO increased the LPO concentration in cancer cells, and induced ferroptosis in them. In addition, excessive ROS also damaged the mitochondria, inducing mitochondria-mediated apoptosis. We evaluated the change of mitochondrial membrane potential (MMP) using the JC-1 probe which changed color from red to green in cells in the presence of damaged mitochondria. As presented in Fig. 5G and Additional file 1: Fig. S9A, MCF-7 and 4T1 cancer cells incubated with pLMOFePt-TGO showed the strongest green fluorescence and the weakest red fluorescence as compared to other groups, indicating a significant reduction of MMP. In addition, western blot analysis revealed the upregulation of Bax expression, and down-regulation of Bcl-2 expression in the pLMOFePt-TGO group, further confirming the occurrence of mitochondria-mediated apoptosis (Fig. 5H). Moreover, we found that the expression of vascular endothelial growth factor A (VEGF-A) in cells treated with pLMOFePt-TGO was significantly down-regulated. Besides, we also further investigated the anti-angiogenesis effect of pLMOFePt-TGO by tube formation assay of C166 endothelial cells. As shown in Additional file 1: Fig. S10, pLMOFePt-TGO significantly blocked tube formation of C166 cells, and showed concentration-dependent antiangiogenic potential. This result further demonstrated excellent anti-angiogenesis of pLMOFePt-TGO. These results demonstrated that the anti-cancer mechanism of pLMOFePt-TGO was also involved in mitochondria-mediated apoptosis and anti-tumor angiogenesis.

### In vivo* anti-cancer efficacy*

**Fig. 6:**
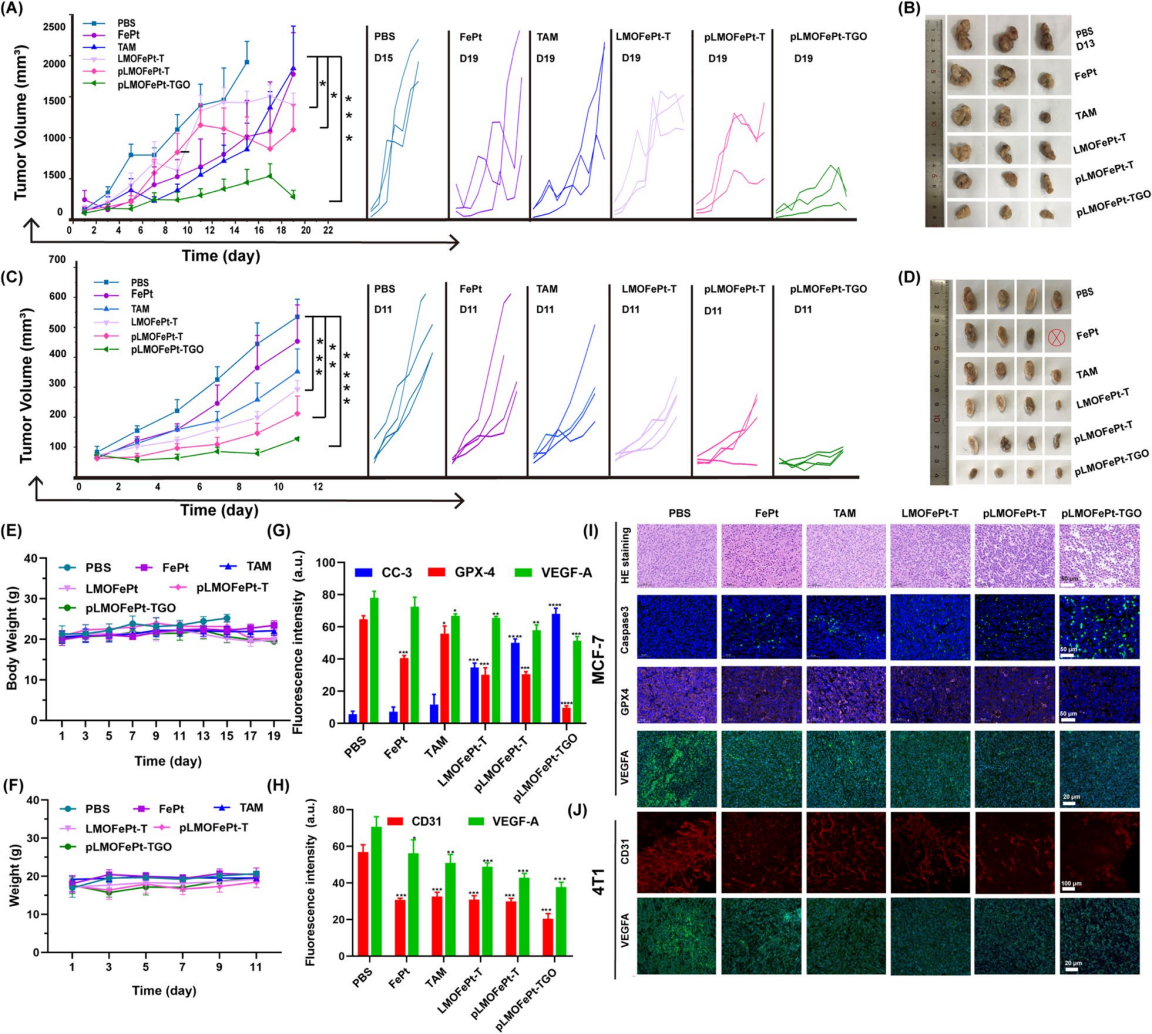
Tumor volumes and photos of MCF-7 tumor and 4T1 tumor **A**–**D** bearing mice injected with different samples at the injection dosage of 2 mg/kg, ⊗ represents death. **E** Body weight changes of MCF-7 tumor-bearing mice treated with different samples. **F** Body weight changes of 4T1 tumor-bearing mice treated with different samples. The corresponding fluorescent quantification analysis for IF staining images of **G** MCF-7 tumor and **H** 4T1 tumor slices. **I** IF and H&E staining images of MCF-7 tumor after treatment. **J** CD31 whole mount staining and IF images of 4T1 tumor after treatment. (mean ± SEM, *Means p < 0.05, **Means p < 0.01, ***Means p < 0.001)

The in vivo anticancer efficacy of pLMOFePt-TGO was investigated by MCF-7 and 4T1 tumor-bearing mice. All animal experiments were approved by the Research Institute Ethics Committee of Binzhou Medical University and were conducted according to the guidelines on the use and care of laboratory animals at Binzhou Medical University. The tumor-bearing mice were intravenously injected with PBS, FePt, TAM, LMOFePt-T, pLMOFePt-T, and pLMOFePt-TGO (2 mg/kg) at two-day intervals. As shown in Fig. 6A, all the treatment groups showed different degrees of inhibition on MCF-7 tumor growth as compared to the PBS group. Moreover, it was found that the tumor volume of PBS-treated MCF-7 tumor-bearing mice increased to approximately 2000 mm^3^ in 13 days. The PBS-treated MCF-7 tumor-bearing mice were euthanized after 13 days, and the tumors were excised. The tumor volume of MCF-7 tumor-bearing mice injected with pLMOFePt-TGO was the smallest among all the groups in this study, indicating it had the strongest anti-cancer activity. After treatment for 19 days, the mice in other groups were also euthanized, and the tumors were excised and photographed (Fig. 6B). The pLMOFePt-TGO group had the smallest tumor size and least tumor weight. Similar results were observed in the 4T1 tumor model (Fig. 6C, D). This result demonstrated that the anticancer activity of pLMOFePt-TGO was independent of the expression of estrogen receptors in breast cancer. Mice in all the groups had no significant body weight loss throughout the therapy (Fig. 6E, F). The vital organs and the tumor tissues were cut into slices and stained using the hematoxylin and eosin (H&E) and immunofluorescence (IF) staining method. Moreover, no deaths of 4T1 and MCF-7 tumor-bearing mice were noted in the pLMOFePt-TGO group, and the results in Additional file 1: Fig. S11 showed no damage in major organs, implying excellent biosafety of pLMOFePt-TGO. As shown in Additional file 1: Fig. S12, Fig. 6G, I, the degree of expression of Ki67 in tumors in the various treatment groups was found to be in the following order: PBS > FePt > TAM > LMOFePt-T > pLMOFePt-T > pLMOFePt-TGO, while the degree of expression of cleaved caspase-3 in tumor tissues was in the reverse order. These results demonstrated that pLMOFePt-TGO had the highest apoptosis-inducing ability and permitted the lowest proliferation of tumor cells. In addition, the GPX4 levels detected in the tumor slices in the various treatment groups were the following order: PBS > TAM > FePt > LMOFePt-T > pLMOFePt-T > pLMOFePt-TGO. This result further confirmed the idea that ferroptosis was induced by pLMOFePt-TGO. Furthermore, the pLMOFePt-TGO group had the highest proportion of apoptotic cells and the maximum number of necrotic areas as compared to the other groups. These results amply demonstrated that pLMOFePt-TGO treatment had the strongest anti-tumor activity among the drugs tested owing to the synergistic integration of chemotherapy, CDT, and starvation therapy.

PDGFR-β is known to be overexpressed in most tumor stromal cells and tumor vasculature [30]. Hence, pLMOFePt-TGO might also be expected to target tumor vasculature and inhibit tumor angiogenesis. Moreover, it was previously reported that TAM contributed to chemotherapy as well as downregulated VEGFA and induced anti-angiogenesis [31]. We further analyzed tumor angiogenesis by whole-mount CD31 staining. In Fig. 6J, PBS-treated tumor slices showed a dense vascular network, while the tumor slices of mice treated with other medication showed vasculature that was significantly less dense, and tumor tissues from mice treated with pLMOFePt-TGO showed maximum suppression of tumor angiogenesis. This result proved our hypothesis that pLMOFePt-TGO could directly target tumor vessels and inhibit tumor angiogenesis. The statistical analysis of CD31 fluorescence signal intensity confirmed this conclusion (Fig. 6H). Immunofluorescence (IF) characterization showed that the expression of VEGFA was significantly downregulated in tumors treated with pLMOFePt-TGO as compared to other groups, indicating that pLMOFePt-TGO also inhibited tumor VEGFA expression and caused tumor anti-angiogenesis (Fig. 6G, H). Therefore, we concluded that pLMOFePt-TGO showed excellent tumor anti-angiogenesis via dual-targeting pathways.

### In vivo* MRI studies*

**Fig. 7:**
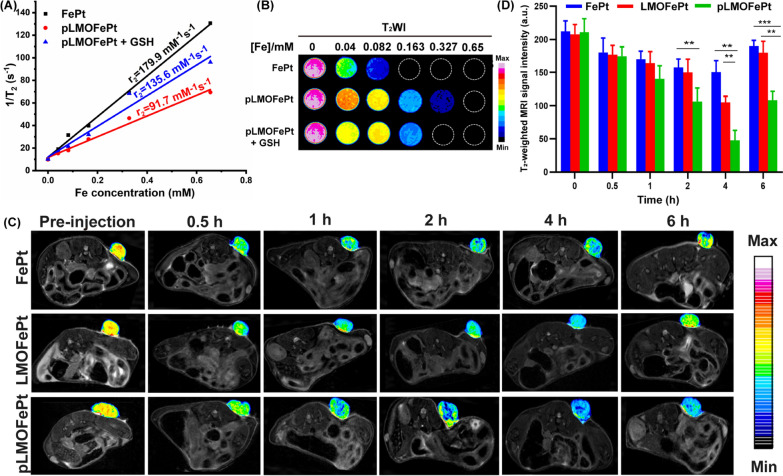
**A** The r_2_ values **B** corresponding pseudocolor T_2_-weighted MR images of FePt alloys and pLMOFePt with and without GSH (10 mM) treatment. **C** T_2_-weighted MR images and **D** corresponding MRI signal changes of tumor at axial plane at post-injection (2 mg/kg) of FePt alloys, LMOFePt, and pLMOFePt (mean ± SEM, *Means p < 0.05, **Means p < 0.01, ***Means p < 0.001)

Owing to the presence of the superparamagnetic FePt alloys, pLMOFePt-TGO could have significant ramifications in magnetic resonance imaging (MRI) diagnosis. Transverse relaxivity (r_2_) measurements of FePt and pLMOFePt were conducted using a 7.0 T MRI scanner. The r_2_ values of FePt alloy and pLMOFePt were 179.9 mM^−1^ s^−1^ and 91.7 mM^−1^ s^−1^ respectively (Fig. 7A, B). The r_2_ value of pLMOFePt was lower than that of FePt, which was attributed to the confinement effect of the organic silica framework. Notably, after GSH treatment, the r_2_ value of pLMOFePt increased to 135.6 mM^−1^ s^−1^, showing a relaxation switch. This showed that pLMOFePt degraded in the TME, releasing FePt alloy, which helped to improve contrast in T_2_-weighted MRI scans. The ability of pLMOFePt to enhance MRI contrast was also explored using a 4T1 tumor-bearing mouse model. T_2_-weighted images of the tumors were acquired at different time intervals (Fig. 7C). The parts of the MRI scan corresponding to the tumor in mice injected with pLMOFePt gradually darkened, indicating that pLMOFePt rapidly accumulated in the tumor. In contrast, the regions in the MRI scans corresponding to the tumors in mice injected with FePt alloys only darkened slightly. This could be attributed to the FePt alloys being easily metabolized, thus impeding their accumulation in the tumor due to the EPR effect. In addition, the changes in the signal intensity of the tumor region were determined using MRIcro software. The minimal T_2_ signal intensity of the tumor region treated with FePt alloys, LMOFePt, and pLMOFePt was observed to reach 150.45, 105.00, and 47.58, respectively (Fig. 7D). Thus, it is evident that among the materials tested, pLMOFePt is also the best at enhancing the contrast of the tumor in T_2_-weighted MRI scans, adding to its potential as theranostic for tumors.

## Conclusions

We successfully fabricated a biodegradable theranostic platform for tumor-targeting CDT that synergistically combines chemotherapy and tumor starvation. This theranostic platform was shown to quickly degrade selectively in response to TME, releasing FePt alloy, GOx, and TAM. In addition, this theranostic platform enhanced cellular acidity and self-supplied endogenous H_2_O_2_ via hypoxic glycolysis and oxygen-consuming glucose oxidation, enhancing the CDT process. In addition, this theranostic platform could specifically recognize breast cancer and effectively selectively accumulate in the tumor, showing excellent targeting ability. The systemic delivery of pLMOFePt significantly enhanced the MRI contrast ratio of the tumor region, making for an easier and more accurate diagnosis. In vitro and in vivo results suggested that pLMOFePt-TGO effectively suppresses tumor growth with low systemic toxicity. Therefore, this work provides innovative ideas for developing multi-modal theranostic platforms for tumor diagnosis and treatment.

## Supplementary Information

**Additional file 1: Figure S1.** A) The hydrodynamic size of FePt, MOFePt, pLMOFePt-TGO. B) Hydrodynamic size change of pLMOFePt-TGO pLMOFePt-TGO in a different medium. **Figure S2.**The particle size and polymer dispersity index (PDI) change of pLMOFePt-TGO in (A) distilled water, (B) PBS, and (C) DMEM with standing time. **Figure S3.** A-F) XPS high-resolution spectra of Fe2p, Pt4f, Si2p, S2p C1s, and full spectra in pLMOFePt-TGO. **Figure S4.** Zeta potential of FePt, MOFePt,pLMOFePt-TGO. **Figure S5.** A) Fourier transform infrared spectroscopy of pLMOFePt-TGO. B) Thermogravimetric curve of TAM, MOFePt, and MOFeP-T. C) The UV-vis standard curve of GOx. D) The loading rate of FePt and GOx calculated by ICP-MS analysis and UV-vis standard curve of GOx in E). **Figure S6.** A) pH value change of different concentration glucose solution treated with pLMOFePt-TGO. B) Cumulative release profile of FePt from pLMOFePt in various pH solutions. The internalization of pLMOFePt in MCF-7 cells: C) low magnification of Bio-TEM observation and D) high magnification of Bio-TEM observation. **Figure S7.** A) Pharmacokinetic curves of FePt and pLMOFePt analyzed by ICP-MS. B) The standard curve of intracellular acidity obtained by fluorescent probe BCECF-AM. C) The fluorescence spectrum of MCF-7 cells stained with BCECF-AM to assess cellular acidity. **Figure S8.** The viability of MCF-7 cells treated with pLMOFePt-TGO at the absence and presence of (A) DFOM, (B) Fer-1, (C) NAC, and (D) Z-VAD-FMK. **Figure S9.** The images of 4T1 cells stained with JC-1 A) and BODIPY B), respectively after treatment with FePt and pLMOFePt-TGO. **Figure S10.** (A) The tube formation of C166 cells treated with different concentrations of pLMOFePt-TGO; (B) corresponding number of tube formation. **Figure S11.** H&E staining images of the vital organs in different treatment groups. **Figure S12.** Ki67 staining images of MCF-7 tumor-bearing mice treated with different samples.

## Data Availability

The authors do not have permission to share data.

## References

[1] Boedtkjer E, Pedersen SF (2020). The acidic tumor microenvironment as a driver of cancer. Annu Rev Physiol.

[2] Chen Q, Liang Q, Sun X, Chen J, Yang Z, Zhao H, Feng L, Liu Z (2017). H_2_O_2_-responsive liposomal nanoprobe for photoacoustic inflammation imaging and tumor theranostics via in vivo chromogenic assay. Proc Natl Acad Sci USA.

[3] Nishikawa M, Tamada A, Kumai H (2002). Inhibition of experimental pulmonary metastasis by controlling biodistribution of catalase in mice. Int J Cancer.

[4] Adam-Vizi V, Chinopoulos C (2006). Bioenergetics and the formation of mitochondrial reactive oxygen species. Trends Pharmacol Sci.

[5] Lin LS, Song J, Song L, Ke K, Liu Y, Zhou Z, Shen Z, Li J, Yang Z, Tang W, Niu G, Yang HH, Chen X (2018). Simultaneous fenton-like ion delivery and glutathione depletion by MnO_2_-based nanoagent to enhance chemodynamic therapy. Angew Chem Int Ed Engl.

[6] Halliwell B, Clement MV, Long LH (2000). Hydrogen peroxide in the human body. FEBS Lett.

[7] Zhang C, Bu W, Ni D, Zhang S, Li Q, Yao Z, Zhang J, Yao H, Wang Z, Shi J (2016). Synthesis of iron nanometallic glasses and their application in cancer therapy by a localized fenton reaction. Angew Chem Int Ed Engl.

[8] Zha IS, Hu X, Hu Y, Wu B, Xing D (2017). Visible light-induced crosslinking and physiological stabilization of diselenide-rich nanoparticles for redox-responsive drug release and combination chemotherapy. Biomaterials.

[9] Circu ML, Aw TY (2010). Reactive oxygen species, cellular redox systems, and apoptosis. Free Radic Biol Med.

[10] Bataineh H, Pestovsky O, Bakac A (2012). pH-induced mechanistic changeover from hydroxyl radicals to iron(iv) in the Fenton reaction. Chem Sci.

[11] Feng J, Hu X, Yue PL (2006). Effect of initial solution pH on the degradation of orange II using clay-based Fe nanocomposites as heterogeneous photo-Fenton catalyst. Water Res.

[12] Liu Y, Wu T, White JC, Lin D (2021). A new strategy using nanoscale zero-valent iron to simultaneously promote remediation and safe crop production in contaminated soil. Nat Nanotechnol.

[13] Liu S, Yu W, Cai H, Lai F, Fang H, Huang H, He J (2021). A comparison study of applying natural iron minerals and zero-valent metals as Fenton-like catalysts for the removal of imidacloprid. Environ Sci Pollut Res.

[14] Zhang R, Feng L, Dong Z, Wang L, Liang C, Chen J, Ma Q, Zhang R, Chen Q, Wang Y, Liu Z (2018). Glucose & oxygen exhausting liposomes for combined cancer starvation and hypoxia-activated therapy. Biomaterials.

[15] Mueller S, Millonig G, Waite GN (2009). The GOX/CAT system: a novel enzymatic method to independently control hydrogen peroxide and hypoxia in cell culture. Adv Med Sci.

[16] Daurio NA, Tuttle SW, Worth AJ, Song EY, Davis JM, Snyder NW, Blair IA, Koumenis C (2016). AMPK activation and metabolic reprogramming by tamoxifen through estrogen Receptor-Independent mechanisms suggests new uses for this therapeutic modality in cancer treatment. Cancer Res.

[17] Rivenzon-Segal D, Boldin-Adamsky S, Seger D, Seger R, Degani H (2003). Glycolysis and glucose transporter 1 as markers of response to hormonal therapy in breast cancer. Int J Cancer.

[18] Jansson S, Aaltonen K, Bendahl PO, Falck AK, Karlsson M, Pietras K, Ryden L (2018). The PDGF pathway in breast cancer is linked to tumour aggressiveness, triple-negative subtype and early recurrence. Breast Cancer Res Treat.

[19] Du S, Yang Z, Lu X, Yousuf S, Zhao M, Li W, Miao J, Wang X, Yu H, Zhu X, Chen H, Shi L, Xu E, Xia X, Guan W (2021). Anoikis resistant gastric cancer cells promote angiogenesis and peritoneal metastasis through C/EBPbeta-mediated PDGFB autocrine and paracrine signaling. Oncogene.

[20] Juliano J, Gil O, Hawkins-Daarud A, Noticewala S, Rockne RC, Gallaher J, Massey SC, Sims PA, Anderson ARA, Swanson KR, Canoll P (2018). Comparative dynamics of microglial and glioma cell motility at the infiltrative margin of brain tumours. J R Soc Interface.

[21] Kadrmas JL, Beckerle MC, Yoshigi M (2020). Genetic analyses in mouse fibroblast and melanoma cells demonstrate novel roles for PDGF-AB ligand and PDGF receptor alpha. Sci Rep.

[22] Zhang YN, Liu L, Li WL, Song TW, Wang P, Sun DX, Huang XD, Qin X, Ran L, Tian G, Qian JC, Zhang GL (2023). PDGFB-targeted functional MRI nanoswitch for activatable T_1_–T_2_ dual-modal ultra-sensitive diagnosis of cancer. J Nanobiotech.

[23] Jia W, Qi Y, Hu Z, Xiong Z, Lu W (2021). Facile fabrication of monodisperse CoFe_2_O_4_ nanocrystals@dopamine@DOX hybrids for magnetic-responsive on-demand cancer theranostic applications. Adv Compos Hybrid Mater.

[24] Theodossiou TA, Yannakopoulou K, Aggelidou C, Hothersall JS (2012). Tamoxifen subcellular localization: observation of cell-specific cytotoxicity enhancement by inhibition of mitochondrial ETC complexes I and III. Photochem Photobiol.

[25] Li M, Shao Y, Kim JH, Pu Z, Zhao X, Huang H, Xiong T, Kang Y, Li G, Shao K, Fan J, Foley JW, Kim JS, Peng X (2020). Unimolecular photodynamic O_2_-economizer to overcome hypoxia resistance in phototherapeutics. J Am Chem Soc.

[26] Feng L, Xie R, Wang C, Gai S, He F, Yang D, Yang P, Lin J (2018). Magnetic targeting, tumor microenvironment-responsive intelligent nanocatalysts for enhanced tumor ablation. ACS Nano.

[27] Huo M, Wang L, Chen Y, Shi J (2017). Tumor-selective catalytic nanomedicine by nanocatalyst delivery. Nat Commun.

[28] Wang M, Wang D, Chen Q, Li C, Li Z, Lin J (2019). Recent advances in Glucose-Oxidase-Based nanocomposites for tumor therapy. Small.

[29] Wang Y, Wei Z, Pan K, Li J, Chen Q (2020). The function and mechanism of ferroptosis in cancer. Apoptosis.

[30] McCarty MF, Somcio RJ, Stoeltzing O, Wey J, Fan F, Liu W, Bucana C, Ellis LM (2007). Overexpression of PDGF-B decreases colorectal and pancreatic cancer growth by increasing tumor pericyte content. J Clin Invest.

[31] Rydén L, Stendahl M, Jonsson H, Emdin S, Bengtsson NO, Landberg G (2005). Tumor-specific VEGF-A and VEGFR2 in postmenopausal breast cancer patients with long-term follow-up. Implication of a link between VEGF pathway and tamoxifen response. Breast Cancer Res Treat.

