# Supplementary material for: Biodegradable nanoplatform upregulates tumor microenvironment acidity for enhanced cancer therapy via synergistic induction of apoptosis, ferroptosis, and anti-angiogenesis
Source: J Nanobiotechnology. 2023 Feb 22;21:59. doi: 10.1186/s12951-023-01814-5 (PMC9945394; doi:10.1186/s12951-023-01814-5)
Supplement: Supplementary file 1 — Additional file 1: Figure S1. A) The hydrodynamic size of FePt, MOFePt, pLMOFePt-TGO. B) Hydrodynamic size change of pLMOFePt-TGO pLMOFePt-TGO in a different medium. Figure S2.The particle size and polymer dispersity index (PDI) change of pLMOFePt-TGO in (A) distilled water, (B) PBS, and (C) DMEM with standing time. Figure S3. A-F) XPS high-resolution spectra of Fe2p, Pt4f, Si2p, S2p C1s, and full spectra in pLMOFePt-TGO. Figure S4. Zeta potential of FePt, MOFePt,pLMOFePt-TGO. Figure S5. A) Fourier transform infrared spectroscopy of pLMOFePt-TGO. B) Thermogravimetric curve of TAM, MOFePt, and MOFeP-T. C) The UV-vis standard curve of GOx. D) The loading rate of FePt and GOx calculated by ICP-MS analysis and UV-vis standard curve of GOx in E). Figure S6. A) pH value change of different concentration glucose solution treated with pLMOFePt-TGO. B) Cumulative release profile of FePt from pLMOFePt in various pH solutions. The internalization of pLMOFePt in MCF-7 cells: C) low magnification of Bio-TEM observation and D) high magnification of Bio-TEM observation. Figure S7. A) Pharmacokinetic curves of FePt and pLMOFePt analyzed by ICP-MS. B) The standard curve of intracellular acidity obtained by fluorescent probe BCECF-AM. C) The fluorescence spectrum of MCF-7 cells stained with BCECF-AM to assess cellular acidity. Figure S8. The viability of MCF-7 cells treated with pLMOFePt-TGO at the absence and presence of (A) DFOM, (B) Fer-1, (C) NAC, and (D) Z-VAD-FMK. Figure S9. The images of 4T1 cells stained with JC-1 A) and BODIPY B), respectively after treatment with FePt and pLMOFePt-TGO. Figure S10. (A) The tube formation of C166 cells treated with different concentrations of pLMOFePt-TGO; (B) corresponding number of tube formation. Figure S11. H&E staining images of the vital organs in different treatment groups. Figure S12. Ki67 staining images of MCF-7 tumor-bearing mice treated with different samples. [file 12951_2023_1814_MOESM1_ESM.docx]

**Supplementary material**

**Biodegradable nanoplatform upregulates tumor microenvironment acidity for enhanced cancer therapy via synergistic induction of apoptosis, ferroptosis, and anti-angiogenesis**

Caiyun Zhang^1^, Peng Wang^1^, Ya’nan Zhang^1^, Pengpeng Lu^1^, Xiaodan Huang^1^, Yinfeng Wang^1^, Lang Ran^1^, Huan Xin^1,2^, Xiaotong Xu^1,2^, Wenjuan Gao^1^, Yu Sun^2,4,5,^*, Li Zhang^3,^* and Guilong Zhang^1,2,^*

^1^ School of Pharmacy, Shandong Technology Innovation Center of Molecular Targeting and Intelligent Diagnosis and Treatment, Binzhou Medical University, Yantai 264003, P.R. China.

^2^ Institute of Aging Medicine, Binzhou Medical University, Yantai, Shandong 264003, China

^3^ Department of Urology, the First Affiliated Hospital of Anhui Medical University, Institute of Urology, Anhui Medical University and Anhui Province Key Laboratory of Genitourinary Diseases, Anhui Medical University, Hefei, Anhui 230022, P. R. China.

^4^ Key Laboratory of Tissue Microenvironment and Tumor, Shanghai Institute of Nutrition and Health, University of Chinese Academy of Sciences, Chinese Academy of Sciences, Shanghai 200031, China

^5^ Department of Medicine and VAPSHCS, University of Washington, Seattle, WA 98195, USA

*Corresponding author.

E-mail address: [sunyu@sibs.ac.cn (Y. Sun),](mailto:sunyu@sibs.ac.cn,) [lzhang@ahmu.edu.cn](mailto:lzhang@ahmu.edu.cn) (L. Zhang), and [glzhang@bzmc.edu.cn](mailto:glzhang@bzmc.edu.cn) (G. Zhang).

***Materials:*** Platinum Bis (C_10_H_14_O_4_Pt), 1,2-Hexadecanediol (C_16_H_34_O_2_), Dialysis Membranes were acquired from Shanghai Yuanye Bio-Technology Ltd (China). Nonacarbonyldiiron (C_9_Fe_2_O_9_), Dibenzyl ether (C_14_H_14_O), Oleic acid, Oleylamine, Glucose Oxidase from *Aspergillus nige,* meso-2,3-Dimercaptosuccinic acid (DMSA), Tetraethyl orthosilicate (TEOS), Triethanolamine (TEA), Bis[3-(triethoxysily)propyl] tetrasulfide (BTES), hexadecyltrimethylammonium toluene-p-sulphonate, Fluoresceinisothiocyanate (FITC), 1-(3-Dimethylaminopropyl)-3-ethylcaibodiimide hydrochloride (EDC), N-Hydroxysuccinimide (NHS), and hydrogen peroxide (H_2_O_2_) were obtained from Aladdin Reagents Ltd (China). DSPE-MPEG2000, HSPC-90A, CHO-HP were purchased from A.V.T. Pharmaceutical Ltd (China). mPEG-NH_2_ (2000) was acquired from Macklin (China). Trimethylbenzene (TMB, BR) was obtained from Sangon Biotech Ltd (China). Deionized water was produced by Heal Force pure water system (CR-SP412, China). Lactic Acid assay kit was obtained from Nanjing Jiancheng Bioengineering Institute. BCECF AM, Hydrogen Peroxide Assay Kit, Reactive Oxygen Species Assay Kit were acquired from Beyotime Biotechnology (China). Antibodies against GPX4, BCL-2, Caspase-3 were acquired from Affinity Biotech (USA). BAX, AMPK, P-AMPK antibodies were obtained from Cell Signaling Technology (USA). ECL chemiluminescence kit was obtained from Biosharp.

***Synthesis of FePt：***Typically, Pt(acac)_2_ (0.1 g) and 1,2-hexadecanediol (0.2 mg) were added into 20 mL of dibenzyl ether in three-neck flask. The mixed solution was stirred and heated to 100 °C for 30 min, and then quickly added with oleic acid (160 μL), oleylamine (170 μL), and Fe_2_(CO)_9_ (0.365 g). Next, the solution was further heated to 300°C and refluxed under nitrogen gas flow for 3 h. Finally, the reaction solution was naturally cooled to room temperature, then washed with ethanol. Finally, the product was dispersed in cyclohexane for further characterization.

***Synthesis of MOFePt：***Firstly, hydrophobic FePt alloys were modified to hydrophilic FePt alloys by ligand exchange. Briefly, 5 mL of NOBF_4_ DMF solution (0.1 mM) was added to FePt cyclohexane, then the mixture was sonicated for 2 min. Subsequently, the supernatant n-hexane was discarded, and the resulting solution was further added with DMSA (0.05 g). After that, the mixed solution was rocked for 40 min at 170 rpm/min. Next, the resulted FePt alloys were washed with twice using deionized water and ethanol, respectively. Secondly, 0.034 g of TEA and 240 mg CTAT were added to 11.5 mL of FePt aqueous solution (1 mg/mL) and stirred gently for 0.5 h at 80°C. Then, the mixed solution of TEOS (1 mL) and BTES (0.8 mL) was added to the resulting solution with vigorous stirring for 16 h. The products were collected by centrifugation (10000 rpm/min, 10 min) and washed three times with ethanol and water to remove the residual reactants, and dried in a vacuum overnight. To remove the surfactant, 50 mg of products were added in 20 mL NaCl/MeOH solution (10 mg/mL) and stirred at 45 °C for 8 h, and this process was repeated three times.

***Fabrication of PDGFB-PEG Polymer:*** 2 mg of PDGFB cycle peptide were dissolved into 5 mL of [dimethyl](D:/%E6%9C%89%E9%81%93%E8%AF%8D%E5%85%B8/Dict/8.10.3.0/resultui/html/index.html#/javascript:;) [sulfoxide](D:/%E6%9C%89%E9%81%93%E8%AF%8D%E5%85%B8/Dict/8.10.3.0/resultui/html/index.html#/javascript:;) solution containing 10 mg of NHS and 10 mg of EDC. Then, the mixture was reacted at room temperature for 2 h under stirring. Next, the 10 mg of PEG-NH_2_ (Mw: 1500) was quickly added to the mixture and continuously stirred for 6 h. The resulted PDGFB-PEG was purified thrzed by deionized water three times in a 1500 KDa dialysis bag to remove DMF and collected the PDGF-PEG in the aqueous phase.

***Synthesis of*** ***MOFePt-T:*** The MOFePt solution (20 mg/mL, 10 mL) was uniformly mixed into TAM/MeOH solution (20 mg/mL, 10 mL). Then, the mixture was rocked for 24 h at 170 rpm/min. After that, the mixture was centrifuged for 10 min at 5000 rpm/min. The product was lyophilized overnight.

***Synthesis of pLMOFePt-TGO:*** Briefly, PDGFB-PEG (1 mL, 0.5 mg/mL), HSPC (1mL, 10 mg/mL), [cholesterol](D:/%E6%9C%89%E9%81%93%E8%AF%8D%E5%85%B8/Dict/8.10.3.0/resultui/html/index.html#/javascript:;) (60 μL, 10 mg/mL), and DSPE (200 μL, 10 mg/mL) were dispersed in chloroform, and the mixture was evaporated to form film at 37^o^C by vacuum-rotary evaporation procedure. Then, the mixed solution (10 mL) containing GOx (5 mg) and MOFePt-T (10 mg) was added to the bottle, and then continuously rotated for 3 min at normal pressure. Afterwards, the resulting solution was further treated with sonication for 10 min. Finally, the product (pLMOFePt-TGO) was collected via centrifugation (14000 rpm/min, 10 min).

***Fenton Catalytic Performance:*** The ·OH generation was detected by colorimetric 3,3',5,5'-tetramethylbenzidine (TMB) and methylene blue (MB) by ultraviolet-visible absorption spectrometer. The MOFePt was immersed in PBS solution at the presence and absence of GSH for 12 h. After that, 200 μL of TMB (0.5 mM) and 30 μL of H_2_O_2_ (30 wt%) were added into the resulting solution. About 10 min, the absorption of solution was recorded using UV-Vis spectrometer at a wavelength of 650 nm. For the colorimetric MB method, the absorption of MB at 650 nm was reduced as the ·OH concentration increasing. The mixture of 100 μL of MB (0.5 mM) and 30 μL of H_2_O_2_ (30 wt%) were treated with different concentrations of FePt alloys under different pH values. After treatment for 12 h, the absorption curves of MB was recorded using UV-Vis spectrometer.

***Cellular Uptake and Location of pLMOFePt:*** MCF-7 cells were seeded in a confocal dish (10000 cells/well, 1.5 mL of DMEM medium) and incubated for 24 h. Then, the cells were treated with LMOFePt and pLMOFePt at the concentration of 40 μg/mL for 4 h. In addition, the cells in other groups were pretreated with amiloride (10 μg/mL) and low temperature (4°C), respectively, and then further treated with pLMOFePt for 4 h. After that, the cells were washed and incubated with Hoechst 33342 (10 μg/mL) in the dark for 20 min, and then washed repeatedly. Finally, the cells were observed by CLSM. To observe time- and concentration-dependent internalization behavior, MCF-7 cells were also treated with pLMOFePt at the same concentration (40 µg/mL) for 3, 6, and 9 h, and at different concentrations (10, 20, and 40 µg/mL) for 4 h. Afterwards, the cells were treated using similar method, and then observed by CLSM.

**Cell Biocompatibility of MOFePt Carrier*：***Cell viability was assessed by a MTT kit (Beijing Solarbio Science & Technology Co., Ltd.) according to the manufacturer’s protocol. For biocompatibility of MOFePt, THLE-3 cells were seeded in 96-well plates at a density of 8000 cells/well and incubated with different concentrations of MOFePt for 24 h. Subsequently, MTT/DMEM (1:9, 200 μL) was added into every well. After incubation for 4 h, the media were removed and 150 μL of DMSO was added to every well. The cell viability was measured by a microplate reader.

**In Vitro Antitumor Efficacy*：***MCF-7 and 4T1 cells were treated with TAM, LMOFePt-T, pLMOFePt-T, pLMOFePt-TGO at different concentrations (equivalent TAM) for 24 h. After that, MTT/DMEM (1:9, 200 μL) was added into every well. After incubation for 4 h, the media were removed and 150 μL of DMSO was added to every well. The viability of MCF-7 and 4T1 cells was measured by a microplate reader.

***Cellular Lactate Content Analysis：***MCF-7 cells and 4T1 cells were pre-seeded in a 6-well plate for 24 h, and then incubated with PBS, FePt (5 μg/mL), and LMOFePt-T (5 μg/mL) for 12 h. The cells were collected by centrifugation at 1000 rpm/min for 10 min. Next, the cells were washed twice with cold PBS and homogenized by an ultrasonic cell disruptor. The lactate contents in cells were measured by a lactic acid assay kit (Nanjing Jiancheng Bioengineering Institute). Finally, the cellular lactate level was calculated by the absorption at the wavelength of 530 nm, according to the standard curve of lactate.

***Cellular pH Level Analysis：***MCF-7 cells were pre-seeded in a 6-well plate for 24 h and then incubated with FePt, LMOFePt, LMOFePt-T, pLLMOFePt-T, pLMOFePt-TGO for 6 h, respectively. Next, the cells were stained with BCECF-AM (2.5 μM) for 40 min. Then, the nuclei were stained with Hoechst 33342 (1 μg/mL) in dark conditions for 10 min. Subsequently, the cells were washed three times with PBS, and observed by CLSM. In addition, the relative fluorescent intensity was quantified via ImageJ software.

***Cellular ROS Detection：***MCF-7 cells were seeded into 6-well plates and then treated with with FePt, LMOFePt, LMOFePt-T, pLLMOFePt-T, pLMOFePt-TGO for 6 h. Subsequently, the ROS contents in cells were probed with DCFH-DA. Finally, the ROS generation in cells were directly detected by CLSM.

***Live/dead Cell Staining：***MCF-7 cells were seeded into 6-well plates (1 × 10^6^ cells/well) and cultured for 24 h. Then, the cells were treated with PBS, TAM pLMOFePt-TGO for 6 h (equivalent TAM: 5 µg/mL). Subsequently, the cells were stained with calcein-AM and propidium iodide according to the protocol (Beijing Solarbio Science & Technology Co., Ltd.). Finally, cellular fluorescence was monitored by CLSM.

***Mitochondrial Membrane Potential（MMP）and Cellular LPO Detection Assay：***For mitochondrial membrane potential measurement, after treatment with different samples, the cells were stained with JC-1 and Hochest33342 for 20 min following the instructions (Beyotime, C2006). The cells were imaged by fluorescence microscopy. For cellular LPO detection, the cells were treated with FePt alloys, and pLMOFePt-TGO, and then further stained with 5 μM of Liperfluo probe (Dojindo, L248) and Hochest33342 (1μg/mL) for 30 min. Finally, the cells were observed by fluorescence microscopy.

***Apoptosis Assay****：*MCF-7 cells were seeded a 6-well plate at the density of 1×10^6^ cells/well. The cells were treated with PBS, FePt alloys, and pLMOFePt-TGO (FePt: 5 µg/mL) for 24 h. After that, the media were removed, and the cells were washed with cold PBS. Then, the cells were stained with Annexin V-FITC and PI for 15 min and then analyzed by flow cytometry.

***Western Blot Analysis：***MCF-7 and 4T1 cells were incubated with PBS, TAM, LMOFePt-T (equivalent TAM: 5 µg/mL) for 12 h, respectively. Then, the cells were harvested with lysis buffer (50mM Tris-HCl [pH 7.4], 150 mM NaCl, 0.1% SDS, 1% NP-40, 0.5% sodium deoxycholate and pre-mixed with protease inhibitor cocktail (B14001, Biomake). BCA Protein Assay Kit (Beyotime Biotechnology) was conducted to detect the concentration of proteins. Then, the proteins were separated by sodium dodecyl sulfate-polyacrylamide gel electrophoresis (SDS-PAGE) and moved to a polyvinylidene fluoride (PVDF) membrane. The membranes were blocked by 5% skim milk. The bands were detected using the following primary antibodies: anti-p-AMPK, AMPK, GPX4, BCL-2, BAX, and β-actin overnight at 4°C. The membranes were incubated with appropriate horseradish peroxidase (HRP)-conjugated secondary antibodies for 1 h at room temperature (RT). Finally, the specific protein bands were captured with an enhanced ECL chemiluminescence kit.

***Anticancer Activity Assessment In Vivo：***BALB/c female mice (four weeks old) were purchased from the GemPharmatech Biotechnology Co., LTD (Nanjing, China). All animal experiments were approved by the Research Institute Ethics Committee of Binzhou Medical University and were conducted according to the guidelines on the use and care of laboratory animals of Binzhou Medical University. MCF-7 tumor model was established by subcutaneous injection of MCF-7 (5 × 10^7^) cells into the right hind legs of BALB/c mice. When the tumor volume is about 100 mm^3^, the *in vivo* experiments were carried out. The tumor-bearing mice were divided into six groups (n=3): (a) PBS; (b) TAM; (c) FePt; (d) LMOFePt-T; (e) pLMOFePt-T; (f) PDGFB-LMOFePt-TGO. The mice were intravenously injected with these drugs (equivalent TAM: 2 mg/kg). Subsequently, the tumor volume and weight of mice were recorded at every 2 day intervals. The mice were sacrificed on day 19 and major organs and tumor tissues were excised for further histological analysis.

For 4T1 tumor-bearing BALB/c mice, 3×10^6^ of 4T1 cells were subcutaneously injected into female BALB/c mice. When the tumor volume is about 60 mm^3^, 4T1 tumor-bearing BALB/c mice were separated randomly into six groups (n=5): (a) PBS; (b) TAM; (c) FePt; (d) LMOFePt-T, (e) pLMOFePt-T, (f) pLMOFePt-TGO. These mice were intravenously injected with these drugs (equivalent TAM: 2 mg/kg). The tumor volumes and weight of mice were recorded every 2 day intervals.

***H&E and IF Staining：***Tumor tissues from MCF-7 tumor-bearing mice were fixed in 4% paraformaldehyde, dehydrated, paraffin-embedded, sectioned 5 mm thick, and then deparaffinized. The slices were stained by hematoxylin and eosin (H&E). Then, the slices were dehydrated and mounted with neutral balsam. For immunofluorescence (IF) staining, the slices were blocked with 5% bovine serum albumin (BSA), incubated with primary antibodies (anti-GPX4, Ki67 and Caspase3, VEGF-A, CD31) overnight at 4°C. Subsequently, the sections were stained with proper secondary antibodies conjugated with Alexa Fluor 488 or Alexa Fluor 594 for 1 h at RT. Finally, cell nuclei were stained with DAPI. These images were photographed by a fluorescence microscope.

***MRI Study In Vitro and In Vivo：***A set of spin echo images of different concentrations of FePt and pLFePt were collected to achieve their transverse relaxation time (T_2_). The transverse relaxation time (T_2_) were measured with a multi-spin echo (MSE) sequence, TR/TE = 5000/10, 20, 30, 40, 50, 60, 70, 80, 90, 100 ms; FOV=120 mm×120 mm; Matrix size=240×240; slice thickness=3.0 mm (5 slices, gap=0). Anatomical T_2_-weighted (T_2_w) images of mice were acquired in the sagittal orientation using Turbo Spin Echo (TSE). The detailed parameters were as follows: repetition time (TR) = 370 ms, echo time (TE) =11.6 ms, field of view (FOV) = 35 mm×35 mm, matrix size = 256×256, slice thickness = 0.8 mm (16 slices, gap = 0), 1 average, and bandwidth (BW) = 50 kHZ.


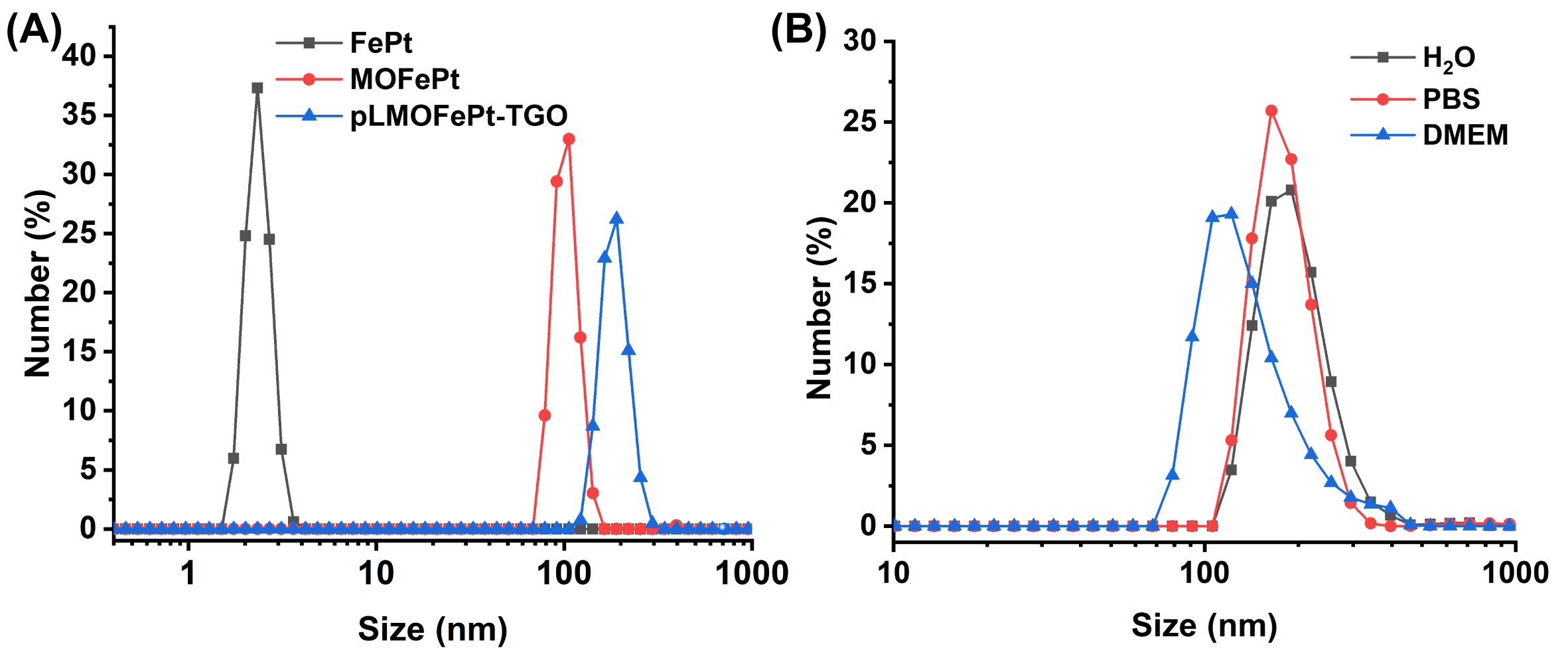


**Fig.S1.** A) The hydrodynamic size of FePt, MOFePt, pLMOFePt-TGO. B)Hydrodynamic size change of pLMOFePt-TGO pLMOFePt-TGO in a different [medium](D:/%E6%9C%89%E9%81%93%E8%AF%8D%E5%85%B8/Dict/8.10.3.0/resultui/html/index.html#/javascript:;).


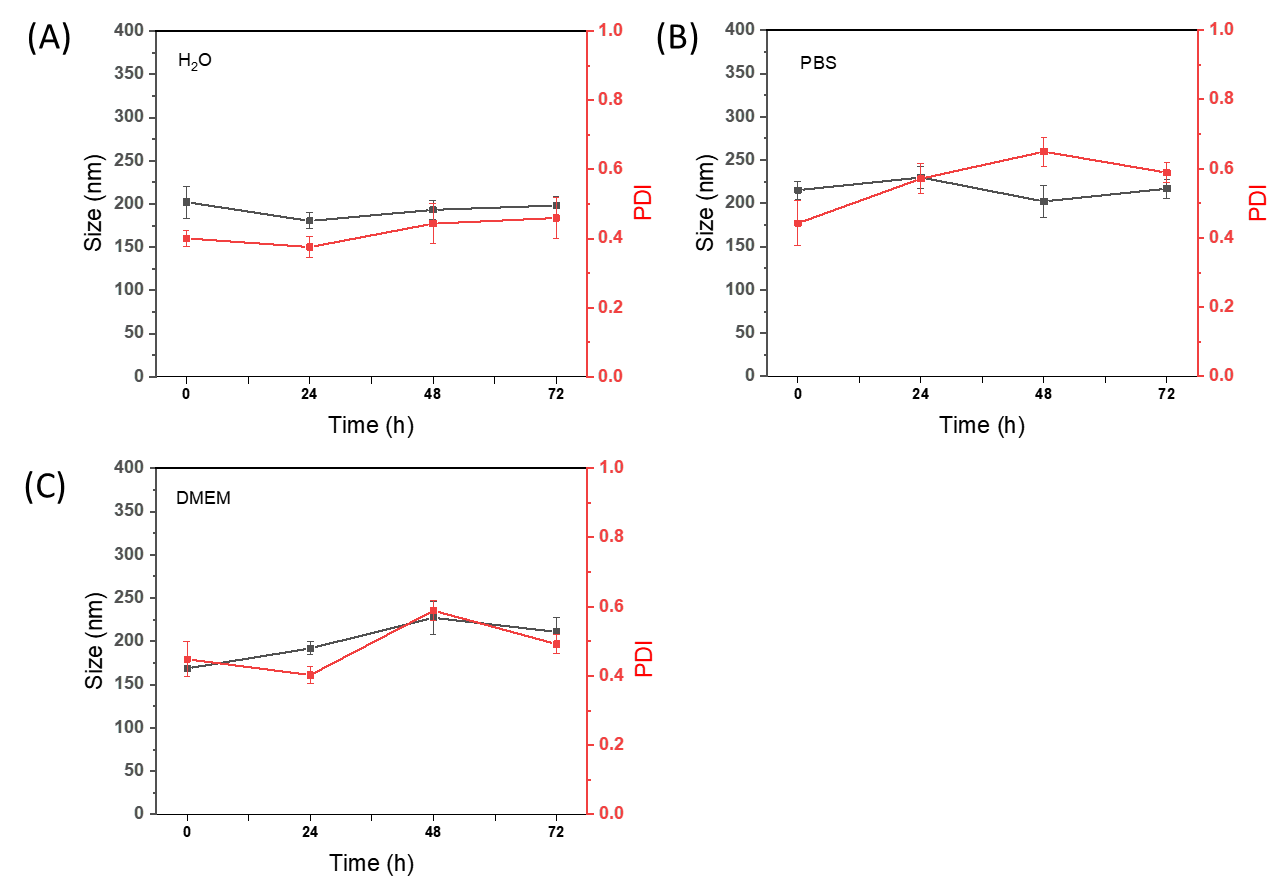


**Fig. S2.** The particle size and polymer dispersity index (PDI) change of pLMOFePt-TGO in (A) distilled water, (B) PBS, and (C) DMEM with standing time.


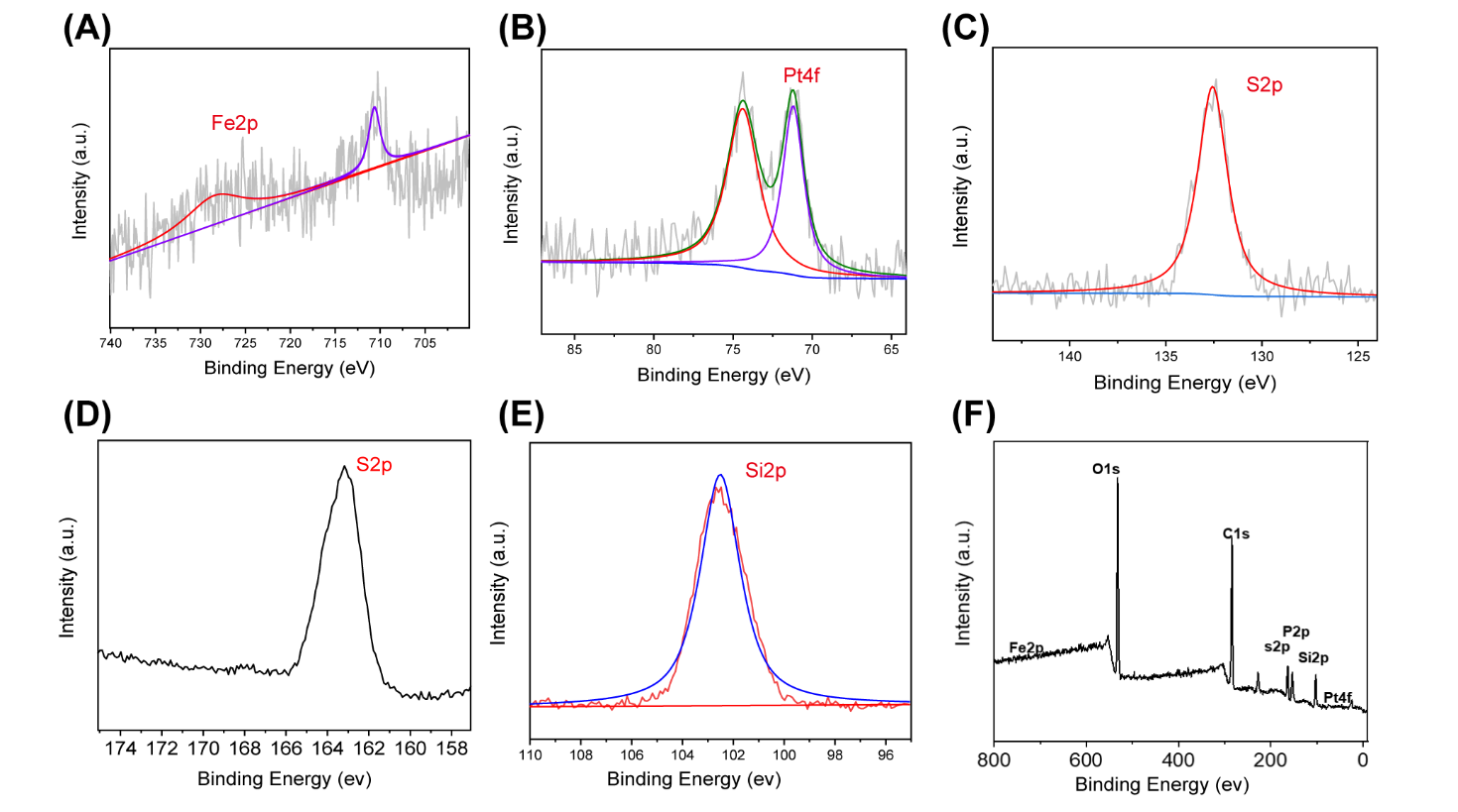


**Fig. S3.** A-F) XPS high-resolution spectra of Fe2p, Pt4f, Si2p, S2p C1s, and full spectra in pLMOFePt-TGO.


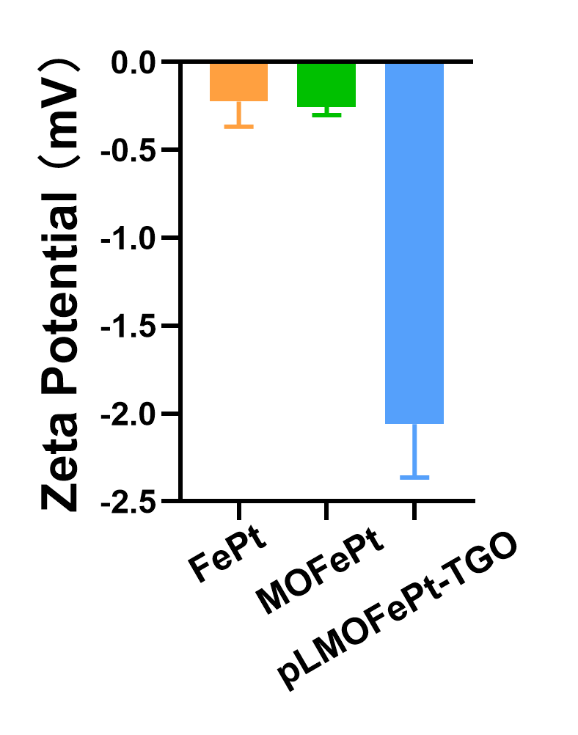


**Fig. S4.** Zeta potential of FePt, MOFePt,pLMOFePt-TGO.


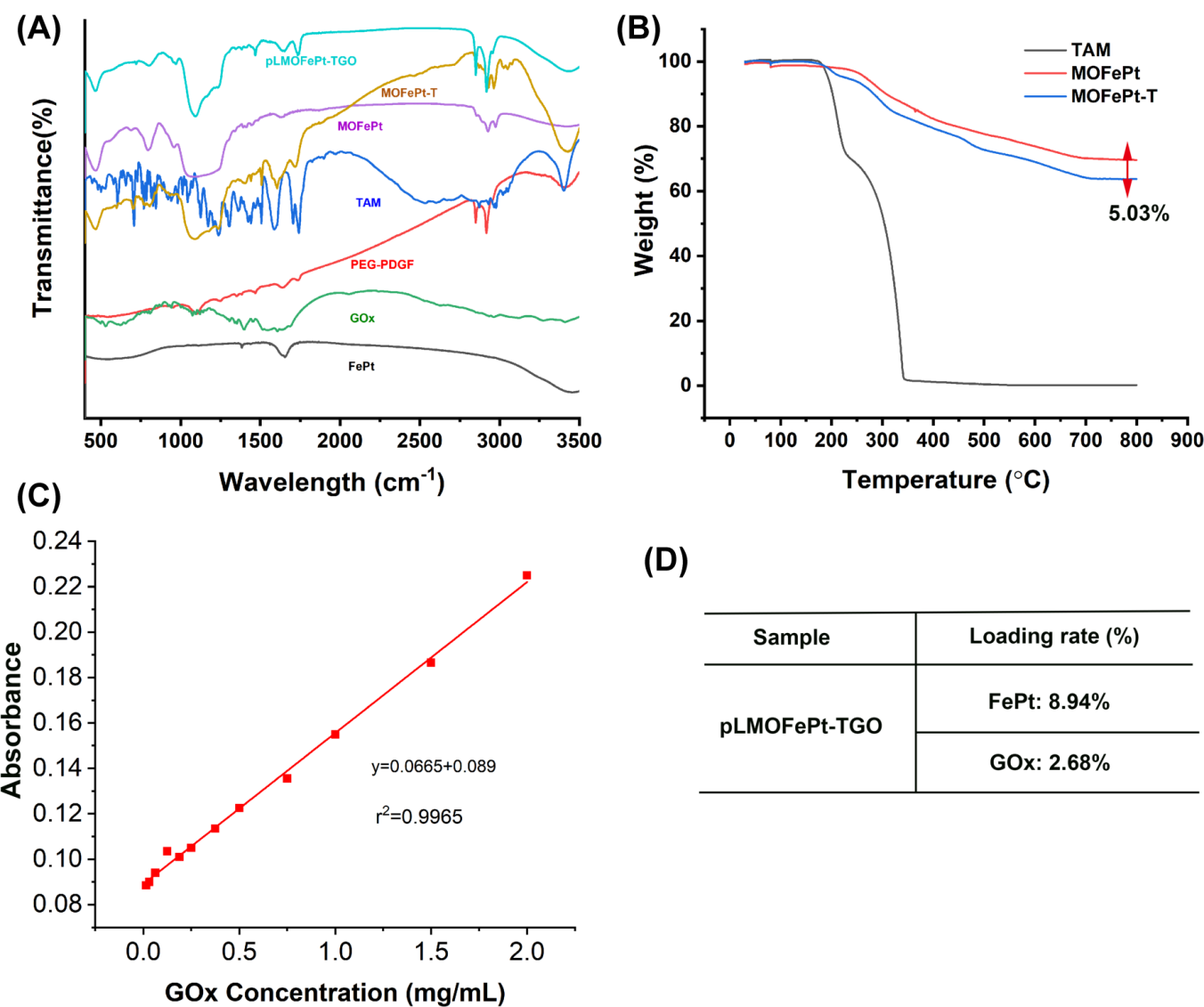


**Fig. S5.** A) Fourier transform infrared spectroscopy of pLMOFePt-TGO. B) Thermogravimetric curve of TAM, MOFePt, and MOFeP-T. C) The UV-vis standard curve of GOx. D) The loading rate of FePt and GOx calculated by ICP-MS analysis and UV-vis standard curve of GOx in E).


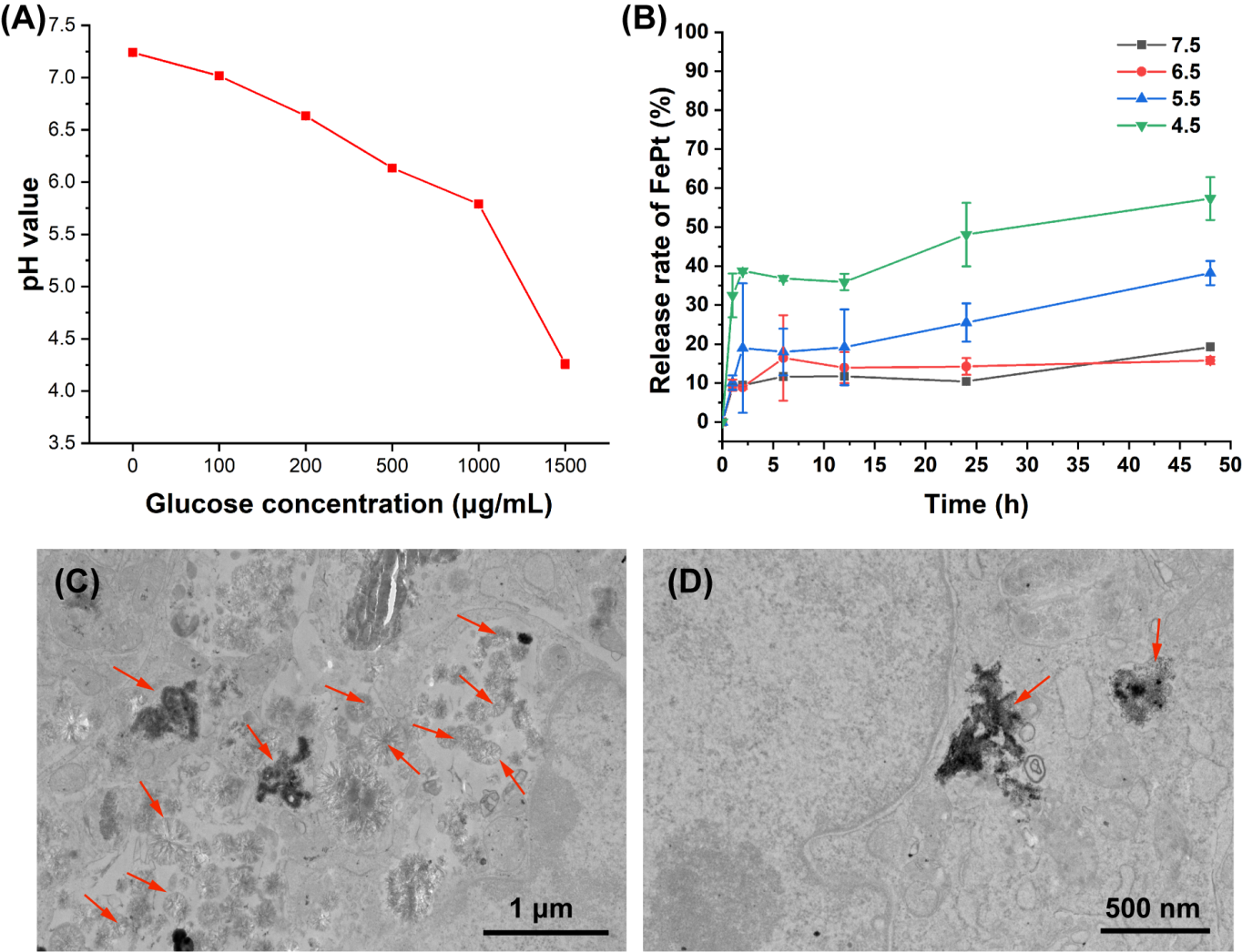


**Fig. S6.** A) pH value change of different concentration glucose solution treated with pLMOFePt-TGO. B) Cumulative release profile of FePt from pLMOFePt in various pH solutions. The internalization of pLMOFePt in MCF-7 cells: C) low magnification of Bio-TEM observation and D) high magnification of Bio-TEM observation.


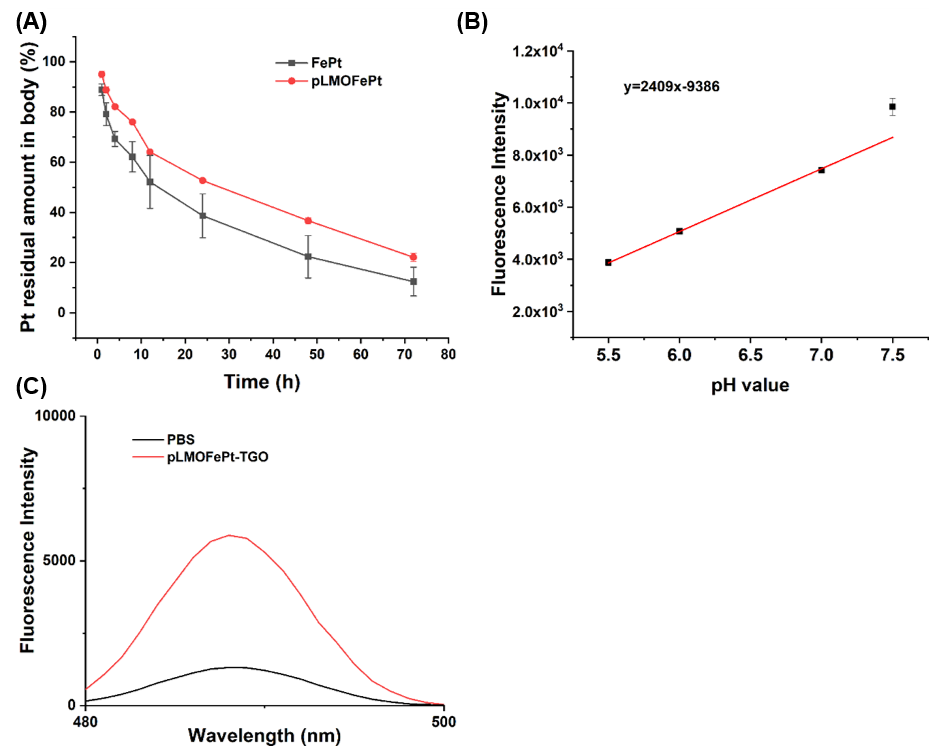


**Fig. S7.** A) Pharmacokinetic curves of FePt and pLMOFePt analyzed by ICP-MS. B) The standard curve of intracellular acidity obtained by fluorescent probe BCECF-AM. C) The fluorescence spectrum of MCF-7 cells stained with BCECF-AM to assess cellular acidity.


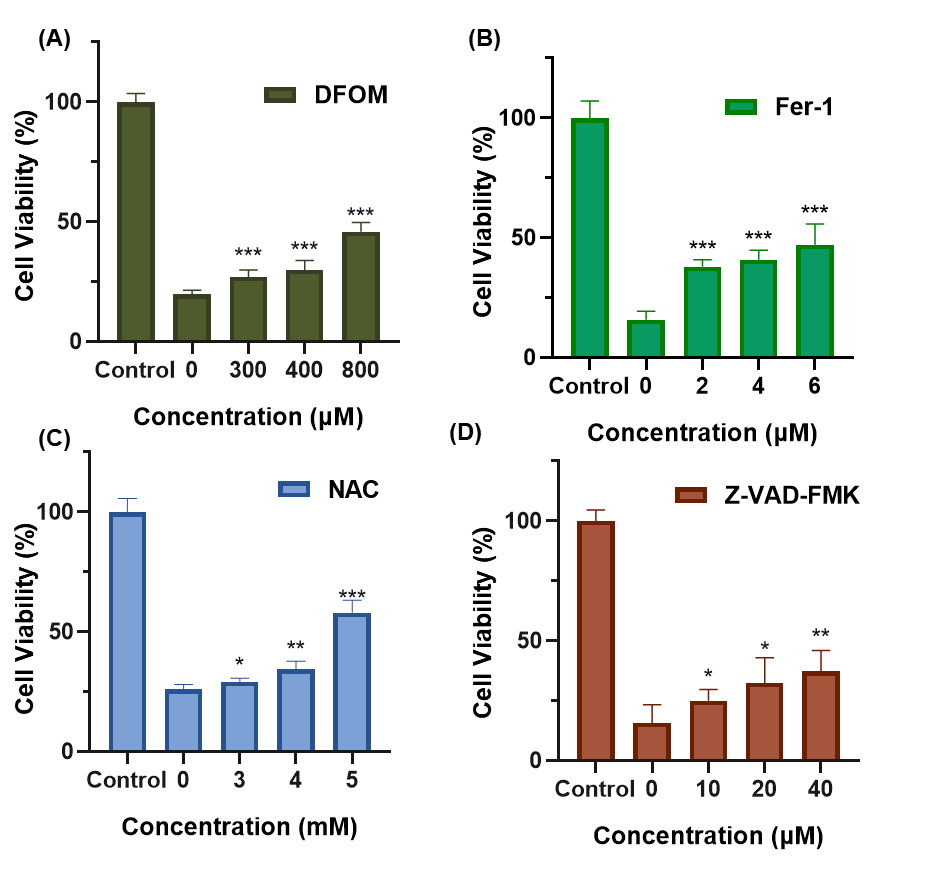


**Fig. S8.** The viability of MCF-7 cells treated with pLMOFePt-TGO at the absence and presence of (A) DFOM, (B) Fer-1, (C) NAC, and (D) Z-VAD-FMK.


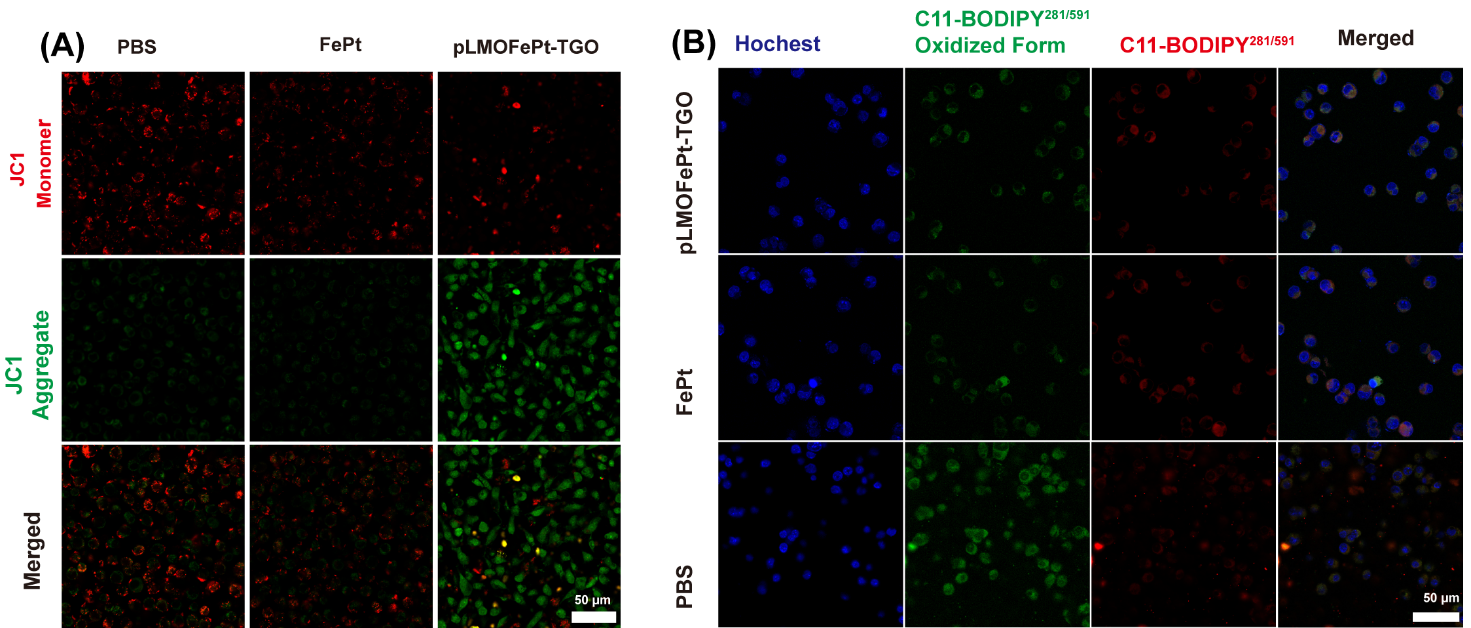


**Fig. S9.** The images of 4T1 cells stained with JC-1 A) and BODIPY B), respectively after treatment with FePt and pLMOFePt-TGO.


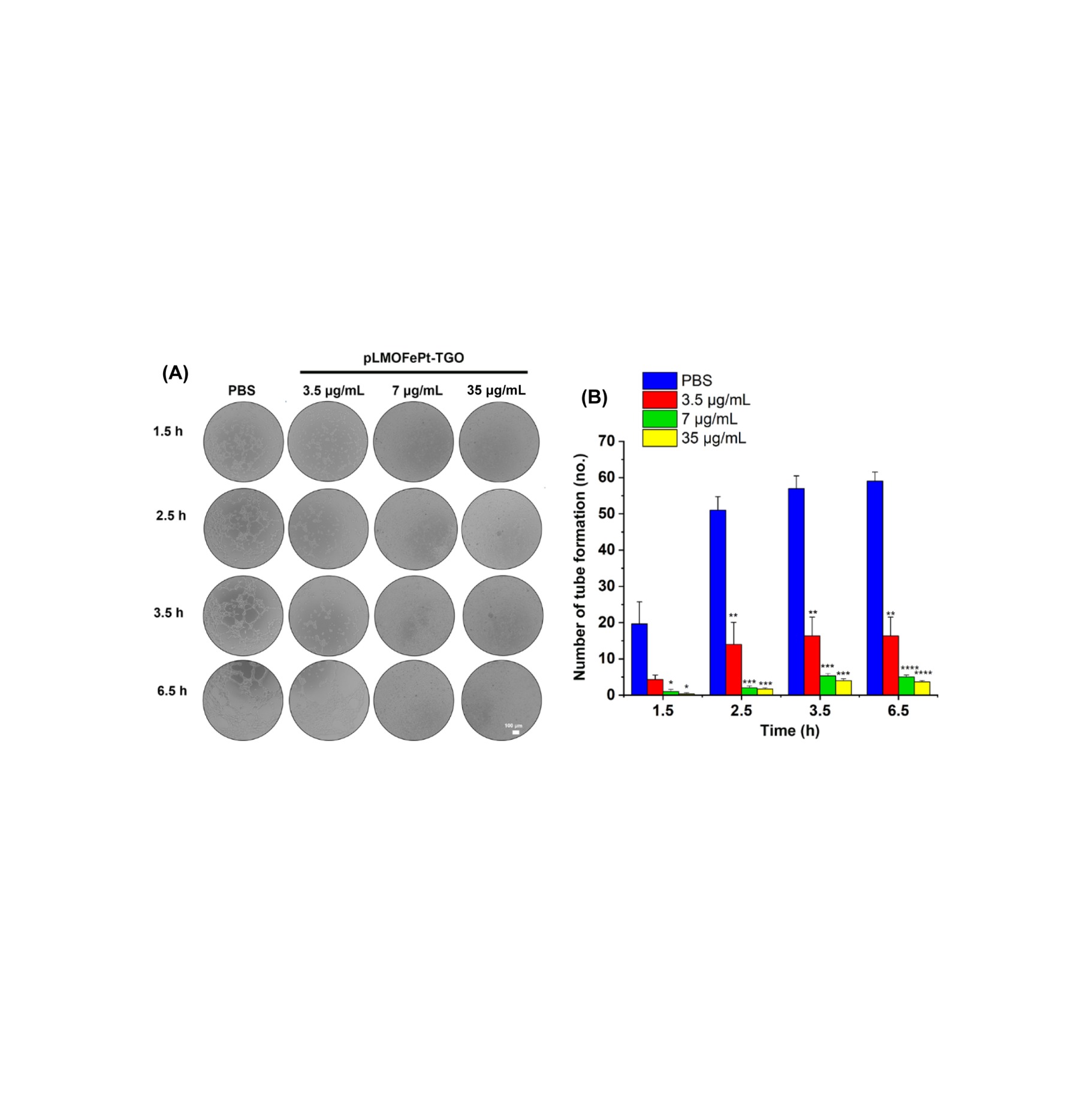


**Fig. S10.** (A) The tube formation of C166 cells treated with different concentrations of pLMOFePt-TGO; (B) corresponding number of tube formation.


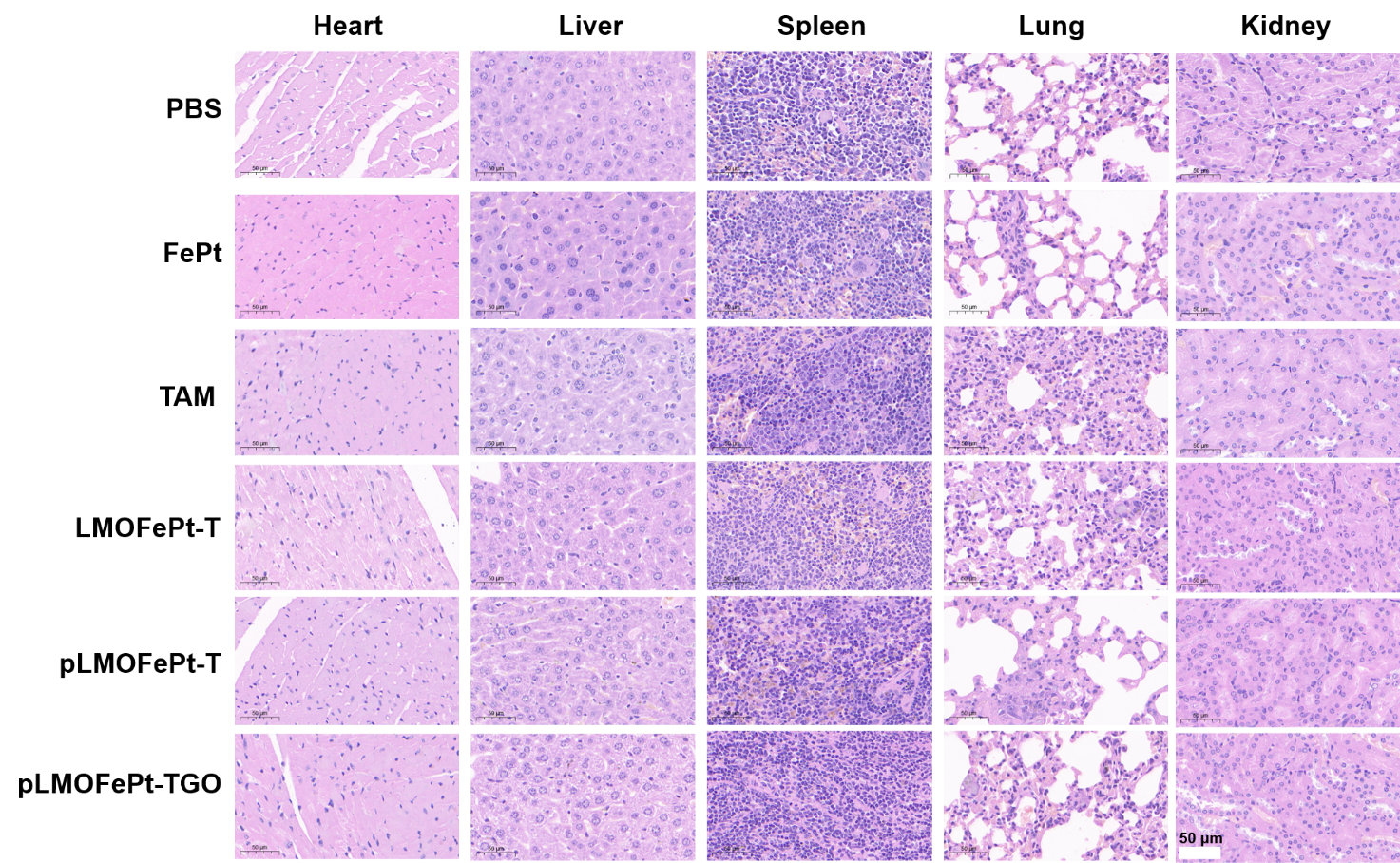


**Fig. S11.** H&E staining images of the vital organs in different treatment groups.


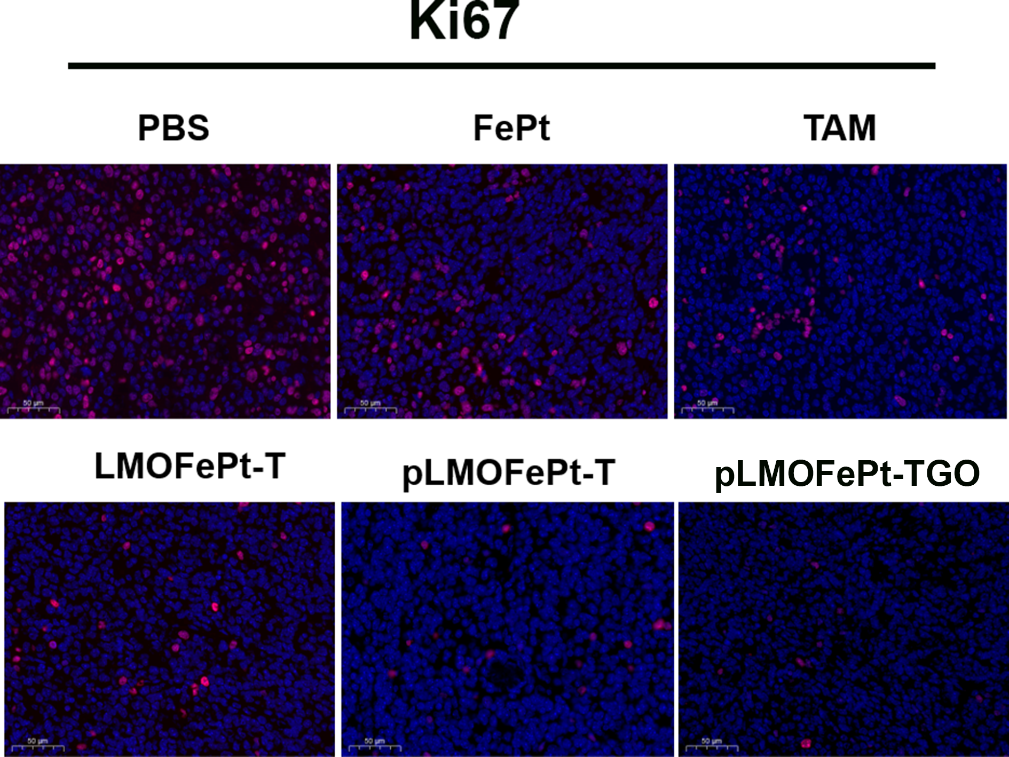


**Fig. S****12.** Ki67 staining images of MCF-7 tumor-bearing mice treated with different samples.
